# Supplementary material for: Lived experiences and drivers of induced abortion among women in central Uganda
Source: PLOS Glob Public Health. 2023 Dec 6;3(12):e0002236. doi: 10.1371/journal.pgph.0002236 (PMC10699625; doi:10.1371/journal.pgph.0002236)
Supplement: S1 Text — (DOCX) [file pgph.0002236.s002.docx]

## **S1 Text: Excerpts from the transcripts**

1. TRANSCRIPT NAME: Participant 11_Rakai, age 25

You said that her boyfriend has to get to know about it so that they can agree upon what to do, how about other people?

Even these elder persons suppose she wants to use herbs, sometimes she may fear to go there and talk to the old people , so in so doing they get to know about it.

Amongst your close friends, how many do you know that they have ever terminated a pregnancy?

I know of only one friend of mine.

So how did you start getting to know about it?

When they were discussing about it that our friend had terminated a pregnancy, that’s when she told me that I terminated it but was greatly affected. Then I asked her why? Then she told me that “No, she might not have terminated it. She told me in that kind of situation.

But she didn’t come the other time before.

No she had already done it.

You said that you terminated your pregnancy in February this year; is that right?

Yes.

And it’s the only pregnancy you have terminated?

Yes it is the only one.

I would like you to tell me how the whole process was from the time you found out that you were pregnant up to the time how you terminated and when you got better.

I conceived around September 2020 because by the time I aborted in February it was almost making five months. Honestly, I really did not want to because I conceived willingly. But when I conceived we got serious misunderstandings with my husband. The situation was not good, the man was a teacher, from Mbale [Eastern Uganda]. When we had misunderstandings, it seems he had issues at his workplace too, so he left the job and went back to his birth place. He did not call, and when I called, he did not answer and eventually blocked my number. I then used my friend’s phone to call him but whenever he heard my voice, he switched off. He left knowing I was two months pregnant because we did a pregnancy test at a clinic together. I imagined how to survive in such a condition. Then I thought of aborting when he left, but I thought that he will change his mind because there was no reason not to. For example like may be that the woman annoyed me but even if she annoys you and you know that she is pregnant, you come back to your senses and continue supporting her but he didn’t do it. I was terminated from work too while pregnant. So, I asked myself, “What I am I going to do with the pregnancy?” I don’t know the home of the man or any of his relatives. Even if I call, he does not care. I was forced to abort.

The fact is that I didn’t have enough money so I decided to terminate it. I went to a health worker but before that time whenever I felt like terminating it in my heart would say please leave it yet it continued to grow and the man’s manners continued to get worse actually he eventually put his phone off. I finally decided to terminate the pregnancy.

When I went to the healthy worker, I didn’t have money I had 60,000/= so she gave me a pill to insert in my private part and another one to be put under the tongue. When I did what she told me I had severe abdominal pains yet I was not bleeding but had pain in the abdomen. I went back and told her that I have too much pain yet it is now the second day and it’s not coming out. So the health worker gave two more pills for swallowing, another one to insert into the vagina and one to be put under the tongue. These quickened the process because as soon as I reached home I started bleeding but the thing (fetus) inside was not coming out for about four days.

At some point I was so scared thinking that honestly I am going to die, I have nothing to do, I no longer have money to go to the hospital. I was scared of going to the hospital because the health workers would abuse me or even chase me, so what would I tell them because they will realise that I was trying to have a pregnancy termination, how will I start?

There is an old woman in the village who gives local herbs so I thought about going there and informing my mother too. When I went and told her thinking that she would be merciful to me and give me some money so that I could go to the hospital, I explained to her everything the way it had happened but she didn’t bother to listen to me; she just stood up and asked me, “don’t children without their fathers grow?”

1. TRANSCRIPT NAME: Participant 20_Rakai, age 39

In the previous survey you said that you had also done something to intentionally end a pregnancy. I would like to talk about this recent pregnancy and I want to ask more questions about that. You mentioned that it has happened to you twice.

This last one was worse.

The first incidence happened when?

In 2018

Okay, we shall focus on the recent time, which happened in 2020?

2020

What was the month?

December

What was the period? 4 months?

It was 4 months pregnancy

During that time what was your occupation?

It is the same activity I am doing currently

That poultry keeping and farming. Do you have children in secondly level?

That one who is 17 years, I have one who is 12 years and would have sat PLE, then 7 years in P.1

For how long have you been in the community?

I have been there for over 23 years.

We shall talk about this recent pregnancy termination which occurred in 2020. I would like you to share with me how did you find out that you were pregnant at that time and your experience during and after pregnancy termination. How did you find out you were pregnant?

I missed my periods then I started feeling feverish, so I went and bought the pregnancy test kit, then I tested myself only to find I was pregnant. I informed my husband. Then he said, ‘that is impossible. I am not responsible for that pregnancy.’ The fact was that he was not responsible, I had got it outside of marriage.

Was it a fact? Yes, it was the truth, and most of us women apart from a lady who could have slept with three or four men in a day who may not know the man responsible but for others who could have slept with one man or two she can know that so and so made me pregnant, most of us can understand it. I knew where I had got that pregnancy, I thought it would look weird, maybe I would continue with the pregnancy and give the child to my husband. But already he had denied it, so I decided to have an abortion. During a pregnancy termination I went through a lot of problems. I got severe diarrhea, I developed different diseases, during that process I got diarrhea.

Was it after termination or during termination?

It was after termination, after pregnancy termination I fell sick I started vomiting, developed diarrhea, it was even alleged that for an HIV infected person failure to take drugs or not being enrolled on medication has led to more multiplication of the virus. I vomited, I got fever, my lower limbs got swollen and I could not walk (showed it; starting from the thighs down up to the feet) and I would ease myself on a bucket, I couldn’t do any job. I got so many problems and since that time I do not think I will have any more pregnancy termination.

By making the decision to end this pregnancy did you inform your partner who was responsible?

Who was responsible, a person responsible should be my husband at home, could be responsible for the pregnancy?

Yes, that is it.

Yeah, I told him and he said it is up to you, he asked me, ‘why did you allow to become pregnant?’

1. TRANSCRIPT NAME: Participant 7_Kampala, age 31

This one we once talked about it the other day when we met and talked about it today. Do know of any woman among the people you are usually with or talk with when she has ever terminated the pregnancy?

Yes. My friends I have told you about. The friends I have are many. Therefore, among these I have friends who terminate pregnancies. They come and tell me XXX it is finished [kyawende] even me when I terminate, I go and tell them. These are the people I call friends because that one who tells you about it is a friend. The person who does not tell you about a secret that one is not a real friend - it is mere friendship.

Is there any other way that you knew that they had terminated the pregnancy?

If she is my friend, I able to know in a different. A person may be your friend but when you take long to see her. When you are with your friend, she may say I met this person but she has lost weight. When I did know what happened. Therefore, my friend replies that person you telling me about you mean you had not known that she terminated the pregnancy almost a month ago. That is how I can know about it. Therefore, that is mere friendship I have with that person because she terminates but does not tell you. You may find her and ask her whether she is sick and she replies that she is not. I only know from this friend who has told me about it.

You told me the other day that the last pregnancy you terminated was in 2020 last year. Can you tell me how you knew that you were pregnant?

The way I found out that I am pregnant is; I didn’t not menstruate. Secondly, which is not about menstrual period stopping, when I got pregnant, I did not know. Started getting sickly not understanding what it was about because every pregnancy has its own symptoms. At certain times, I would feel coldness and at other times, I feel not well but I could guess that I was pregnant. I asked myself what was wrong, why I was becoming sickly from time to time. My friend advised that I should go to the hospital but I refused and asked her why. She told me that it is possible that I got pregnant and reply to her that no, a mature woman like me, it is impossible. She told me that the other time when you terminated it were you not mature. This friend of mine knows me very much. I insisted that it is impossible this time - I cannot believe it. I realized that my menstrual period had stopped yet it was supposed to have occurred on the 26^th^ of that month. I thought maybe I missed my period naturally because I have ever done it before. However, I realized missing the periods even in the following months – making it two months. I went to the hospital and by the second month, I had stopped becoming sickly from time to time. In the hospital, the man who attended to me asked that do you think you are pregnant. I replied that I am not and he asked whether, I was sure. I became doubtful so I went and brought the pregnancy test, which confirmed the pregnancy. I called that hospital man [responsible partner] to share with him. We had sex but I did not know that I had conceived. When I called and shared with him, he stopped receiving my calls. Even when I used a different phone number and he recognizes my voice, he would end the call. I asked myself ‘what should I do?’ I realized the pregnancy was growing and I decided not to keep this pregnancy when I am renting. I went and bought the tablets and terminated because I had no other solution

So, after terminating it….

Definitely, I went to my friend like I always do and shared with her that it is finished [ekintu kyawende]. However, I had a bad experience with termination because they had to wash my inside (cleanse). That termination was very bad.

Where did buy the tablets

From the pharmacy just around there. That side of Eden, there are a number of pharmacies close together. That is where I bought the tablets at fifteen thousand shillings.

Who directed you?

My friend I talked about from Nabweru took me there. She took me there directly and told me that such tablets are not sold to anyone and the cost is high. I stayed somewhere and she went brought them to me.

She went by herself.

We went the two of us and she told me those tablets are sold anywhere. It is not easy. Yet for her the person she was getting them from is the one she usually goes to. She got them and we came back home. Because she told me that, it appears some of the pharmacies do sell those tablets.

When you found out that you are pregnant, how did you feel?

I felt bad and worried because I will be the father at the same time the mother of the baby. I asked myself what will I do? You have a pregnancy and the same time they demanding house rent. What will I do to feed myself? However, when you are not pregnant you can decide to go to the bar. On the other hand, when you pregnant everything comes to a standstill.

Now where are the children?

My children are at their father’s home

Okay. you were worried on knowing that you were pregnant

Yes, I did and I settled after it getting out because I couldn’t stay with my pregnancy, I pay rent, no one to give me money for food

Apart from being a father as well as a mother, what other reason made you terminate the pregnancy

Another reason is that I cannot have a child without its father. That is the reason because if a child has no clan is a problem because I myself speaking I am a witness. I do not have a clan and then I give birth to child without a clan. It is very bad for me a grown up, it haunts me a lot and I again give birth to a child without a father

What other reasons apart from those two?

Another reason is that you cannot give birth when you do not have the means of looking after myself. Every person needs a person looking after him/herself. You have a child; they need money for food, rent. If you have the capacity of looking after yourself that is okay, you can give birth to that child. I am unable to look after a child.

Is there another reason?

No. The reasons are that of not having a father and the financial means of looking after the child.

About the tablets. Do you know them?

I have never paid attention to how they are called because the first time of terminating the pregnancy, I used machines. May be if I first make a call to my friend and ask her how the tablets are called. When you have somebody to run to for assistance and you get the tablets, you may not mind much about how they are called.

1. TRANSCRIPT NAME: Participant 19_Rakai, age 25

Generally who do you confide in about private, personal information in general?

I have a friend I am proud of, I can tell her about anything. I don’t have any sibling I confide in but I have a close friend I confide in, she can keep all my secrets.

No relative

No

How about health workers?

I don’t have any friend who is a health worker

How about among your teachers?

No I don’t have any.

How old is your friend?

She is about 32 years old and she is married.

If a woman had a pregnancy termination, who in her life would be most likely to know about it and why?

Majority inform their friends because she might be still living with her parents and she doesn’t want them to know about it. If she does not tell her friend then she will tell the person responsible for the pregnancy. Those two are most likely to know unless the parents… I don’t how to term it because parents are usually so strict about that incidence especially for the unmarried.

There are some married women who terminate pregnancies, in that instance she can discuss with the parent and tell her, “according to how the husband treats me, yet I am having un-spaced children, what can I do?” The mother then can share a plan with her. But if you are not married then you cannot share such information with your parents but with your friends. (Interruption)

We were talking about those whom a woman can talk to.

Okay you might have talked about this a bit during the survey you participated in but I would like to know if you know any woman in your social circle who has ever terminated a pregnancy?

My friend and one of my siblings because I used to stay at her home.

How did you get to know about your friend’s pregnancy termination?

She came and told me so I went with her to the Doctor. She used to stay at her parents’ home so I think they must have learned about it so they supported her to improve.

What was her experience like, how she conceived, the reason for termination?

The man responsible for the pregnancy was married and old so she was not in love with him. It was a relationship whereby one thinks she is extracting money from this man and he ends up pregnant. The man too was not willing to have this baby so they had a discussion, he gave her money and we went to the Doctor at the health facility and bought some medicine.

How about your sister?

I didn’t get all her details but I used to stay with her so I observed her movements, she was not settled and when the pain intensified she had to tell me because I was the only person available. She said, “I was about to die due to this pregnancy termination”. She did not tell me why she had done it but told me that she had terminated a pregnancy which was about to kill her because I was the only person at home. She would tell me to go the shop and buy anything she wanted like a drink, maybe to the health facility to buy some tablets, I supported her with all that but she had not opened up about anything before that so I didn’t know about that.

From your observation, how was her experience like?

It was so bad, her life was at stake and she was wailing in pain. It seems she had visited the Doctor because the following morning she said she had to visit a Doctor. She had called the same person at night who told her to wait until the following morning so when she gained some strength she went back to that Doctor.

During the previous survey you said that you had done something to intentionally end a pregnancy. When was that?

It was 2017 I think in October towards December.

Please tell me about that time from when you conceived, what you did up to the time you got better.

About the pregnancy termination I had? I was still in school and it was a teacher responsible. I was in boarding school when I conceived, about to be promoted to senior four. When I informed the teacher he didn’t want my family members to know, so he took me from school to a doctor in town who was well known to him. That is the doctor who attended to me. He used some equipment. When we left that health facility my health condition got worse, I was so ill, the bleeding was so heavy. This was a boarding school so it was only him who knew about it, okay some other friend too was aware because we used to spend most of the time together so I would tell her about everything. This girl would not support me so much because we were at school and the teacher too didn’t have to show that he knew about what was happening since he had to protect his job. At this time my condition became worse, I was so ill that I had to be taken back home.

When I got home my mother too got to know about it because of my health condition. She supported me buy getting me some herbs, I had some tablets too which were given to me when I went to the health facility so I would use the herbs as I took the tablets too. I eventually improved but this took some time, it was a very bad experience.

You said when you went to the health facility the Doctor used some equipment, did you feel everything he was doing?

The lower part was sedated and the upper part was active but again I would feel some pain, I was not sedated completely for me not to feel any pain, I could feel some slight pain.

You said when you left the health facility…

We went back to school but the situation got worse when we got there. The bleeding was so heavy, I had access to drinks, food but the condition was not good, the bleeding was so heavy and I was so weak too. I had fever, heavy bleeding and this took about four days.

Was the school nurse aware?

No because we didn’t have one.

Who provided health care while at school?

As we left the health facility I was given some tablets and the teacher kept on giving my friend juice and what to eat, I took the tablets but the situation was worsening that’s why I was taken home. He used to buy passion fruits, quencher so that’s what I used to take because I had been advised not to take soda.

How about things like sanitary pads?

I had the sanitary pads because as we go to boarding school such items are packed for us to keep. I had such items.

What care did the teacher provide? How did he take care of you?

He would meet my friend and tell her to bring me those different items and ask if I had taken the medication. Since she was my friend.

Did other students get to know about it?

There are some who got to know because as I was leaving some were wondering what the problem could be, some said but on such a day she wasn’t around. And since you can easily tell people who are in love some students got to know, when I got sick they started asking so many questions, they followed up the story so that’s how some got to know while others didn’t get to know.

How did you feel about the students getting to know?

I felt bad but I didn’t go back to that school

Just to take you a little but back, how did you inform your partner that you were pregnant?

When I conceived, I would feel cold in the evening, I had headache then whenever I would eat food - I didn’t like the beans so whenever I would try to eat I would vomit then I wondered, “what could it be?” Then we bought strips for testing pregnancy and when I tested the results were positive, I was pregnant so that’s how he got to know too. I think that’s when he got to inform that Doctor from Kyotera so one day he took me to Kyotera.

In your view how did he feel?
…

Who made the decision that you should have a pregnancy termination?

I didn’t like it too but he told me that you have to continue with your education, let us terminate it, you cannot have children you are still young. So we had a discussion and agreed to do it. I was so worried, I could not accept it too, I was saying this cannot happen to me I am not pregnant but when we screened I was pregnant.

From the time you started an affair with the teacher weren’t you doing anything to prevent the pregnancy?

I wasn’t using anything because I didn’t think about it, I didn’t like family planning and at home no one guided me about it, I didn’t know about it.

How about condoms?

I didn’t know about them.

How did you feel when you got to know that you are pregnant?

I was so worried, I had fear thinking, “what if I die”? My major worry was about death, I thought I might die yet people at home didn’t know about my situation so I said, “God will take the final decision”. But I didn’t want my family to learn about it yet it’s what I was avoiding most and even insisting to have it terminated, then I said “if it is to die let me die but my family shouldn’t get to know about it.” This didn’t happen because eventually they got to know about it, they were so angry with me, they didn’t take me back to school and I was fed-up too, I wasn’t fitting anywhere. That is how my education ended.

How did life change after that?

When I left home I was taken to Kampala to work as a house helper but because of the way I was treated I didn’t think about what had previously I wasn’t doing anything, I was abstaining but when I realised that I couldn’t handle that anymore I started using a family planning method but not for a very long time and I think I didn’t count the days properly so I ended up conceiving again but I had the baby.

After how long was it?

It was after sometime, about eight months that I conceived again. Before going to Kampala when I cured I was abstaining and eventually I started using a family planning method although I don’t know what happened, I think I didn’t count the days properly, I was using an injection (injectaplan). I was told it takes three months but it didn’t take the three months for me to conceive and I couldn’t go back…

Go back where?

For a pregnancy termination (laughter) I had to bear with it so I had my baby girl.

How about the baby’s father?

He is on his own and I am on my own too although we communicate, he stays in Lukaya, he calls but not often. The other time before I started work he used to take good care but from the time he got to know that I am working he stopped taking care of me unless I call him about an illness then he can support but with other issues he doesn’t mind.

After the test and you were pregnant how long did you make the decision to terminate the pregnancy?

He made a prompt decision the moment he got to know that we are going to have a pregnancy termination so I accepted because I didn’t want people at home to know about it but it took about two weeks I think he was still connecting with the Doctor or a private place so that no one would know about it. After two weeks he told me that we would go to Kyotera to a Doctor who would render good services. It was a Saturday that we went to Kyotera because we never had classes on a Sunday.

What was your experience within the two weeks?

I was in deep thoughts like; “I could die, on the other hand I was thinking you will not die and you will resume with your education, your family members will not get to know about it.

Were you still vomiting?

I was not eating all the time because whenever I ate I would vomit, I could eat snacks or take lots of fluids because I wouldn’t vomit the fluids but I would vomit out the food.

Apart from the teacher, did you at that time tell anyone else about it?

The only other person was my friend, it was my friend only who got to know about it.

What would she tell you?

She would ask me about the decision that the teacher had made, “be strong you will not die”. I would ask her, won’t I die and she would say “no be strong you will not die”.

Had you shared your plan with her?

Yes I had shared with her all the details about our discussion but my major concern was about dying, I thought I would die. She comforted me.

Is she the one you talked about, your schoolmate?

The one I have currently? No, the other one was different from this one.

You said you didn’t want your family to know, you wanted to go back to school, any other reason as to why you didn’t want to have that child at that time?

During that time I really wanted to study so I couldn’t allow that incidence to hinder my education. I wanted to continue with my education.

You said something that the teacher too had to protect his job…

I didn’t mind about him because he made his personal decision, if he was to say that he rather loses his job but we don’t terminate the pregnancy then we would have done that but since I wanted to study I decided to follow his decision.

Do you hear from him?

I don’t know where he went. I think het left too because when the pandemic started some people went back to their home areas then others especially teachers were involved in other jobs so I am not sure whether he went back to his home or he got another job.

You said you went to the health facility, is there any other place where you went trying to do the same thing? (Terminate the pregnancy)

Before going to the other facility? No we didn’t go to any other place.

How about when you went back home from school?

My mother gave me boiled local herbs which I used to take plus the tablets I got at the health facility.

How long did it take you to get better?

One month, I wasn’t bleeding but I bled for some long time, the first week, second week, third week and by the end of the month I had improved but it took me sometime. The bleeding was on and off, today I am bleeding the next day I am not and the amount had reduced. But it was almost a month, after that I didn’t bleed at all, that’s is when I was normal again.

What challenges did you face during the pregnancy termination?

During that time it was about what people were saying, people get to a time and begin talking about you so I made sure that I stay at home. I would stay indoors for instance I didn’t want some of my friends in the community to get to know so I would read my books pretending that it is fever, I continued reading my books and would request some students from nearby for some notes just to show them that I have to go back to school, I am suffering from fever. I didn’t want them to know what had happened to me.

When you got to the health facility, did the health worker do or say anything that made it difficult for you to continue with the termination?

He said I am going to use some equipment, but you must be strong, then I was wondering how the equipment work, I was in great fear. They [tools] can really scare. Have you ever had tooth extraction? The chairs in the dentist room! So, when I got to this room there was a certain smell, the chairs had a funny shape, you feel as if you are in hell, you feel ‘yes, it is time to die’. The health worker mixed medicine, he was preparing his machines, I was all shivering. When I was sedated with chloroform he took sometime organizing this and that as I was watching, this made me more scared and by the time he attended to me, honestly, I was feeling the [perceived] pain. I would feel the pain. When he was done it took me time to regain my consciousness, I didn’t know what was happening, I didn’t know the road we used to come because the health facility was located here and his clinic was a bit far from the health facility.

When he finished attending to me he told me; “ Please rest for some time then come back up there” but honestly when I got out I couldn’t see the road which we used to come to this place, I didn’t have a phone, it is the teacher who had a phone. I kept on looking, the place seemed very new to me like I hadn’t seen it before. There were two women seated (laughter) so I asked them “where did the man who brought me here go?” But I think since they stay there they must have known what takes place inside. So they told me use that road, you will see the health facility, it is next to a mosque. Honestly, I wasn’t in my normal senses, the fear was too much so I was given direction to where I had come from yet it was during day time. I was so scared, I was scared. ((With tears in her eyes))

Apart from the machines, what else scared you?

Nothing more because I expected whatever happened to happen so I wasn’t scared of any other thing, I was strong but the machines are so scary.

Did you think about changing your mind?

Yes I wanted to change my mind but I imagined that it was a longtime plan, we had a discussion about it, the distance was long to this place, when I thought about that I had no option but to accept, if I was to refuse I would have done so while still the other side so since I made a decision let me be strong. I gained my strength but I still thought of changing my mind. I made myself strong.

How about your friend did she say or do anything that scared you?

After telling her about it? No she was comforting me, she told me, “you will be okay, don’t fear, don’t worry the Doctors are trained and you are not the first person to terminate a pregnancy, you are not his first patient, he attends to many people and he is trained in that.” she comforted me that I would not die, she didn’t tell me anything scary.

How did you feel when she talked to you like that?

I was comforted and had hope of surviving but if she told me that I would have died maybe I would have run away from school but she always had time for me, comforting me.

You said it is the teacher who found the place so you didn’t find any hard time finding a place where to go for the termination?

No I didn’t, he organized everything.

1. TRANSCRIPT NAME: Participant 7_Rakai, age 16

In our previous survey you said that you had done something to intentionally end a pregnancy. I would like to ask you more questions about that pregnancy that you terminated. You will describe how you found yourself pregnant, how you felt during termination at that time and also talk about the time the pregnancy termination was complete.

You said that when was the pregnancy termination?

2019.

In 2019, what was your age by then?

I was 14 years

What was the period of the pregnancy?

I think it was 3 months and a half.

In which class were you by then? Were you in vacation?

We were in holidays by that time it was December.

December holidays, which class?

I was in senior one, I used to have lessons during holidays.

Okay, what happened?

To become pregnant?

Yes.

My father had his best friend who used to come home often, and even stay over. I used to prepare food for them, and mum had already left [separated]… One-day he came home. When I finished washing and prepared food, I went to take a bath, but I found him in the sitting room after. I went to the bedroom to dress up and he followed me. He raped me from the bedroom and left. I was still young [on 14 years].

Did you make any alarm? He caught me with force and stopped me from making alarm. He caught me in the neck and I got scared and kept quiet, he raped me from inside the bedroom. He left me there crying, I was in sorrow. Then dad returned and asked, “why have you slept so early?” it was at 8:00pm then I told him I am sick, so he said, “you dress up and we go to the health facility.” I dressed up and we went, found the nearby clinic closed and already it was late so he told me we shall go back tomorrow. The next day I told him am feeling fine even if we don’t go to the health facility. He asked me, “are you sure?” I said yes. So ignored it, and this man disappeared completely he no longer came home, nor see him anywhere, we wondered his whereabouts, later we ignored it.

Didn’t you go to the health facility yourself?

No, I didn’t go there, I couldn’t imagine that I would even become pregnant. So, after three months I started feeling bad. I was feeling weak, lost appetite, I wanted to sleep all the time. What month? It was in November around 23rd when I learnt I was pregnant. I was in the compound then felt headache, felt dizzy, I collapsed. I gained consciousness while in the school sickbay. Then I asked the nurse what had happened to me. She asked if I had ever had sex. At first, I denied and said no, she said please say the truth, again I denied but she continued asking. So, I narrated to her all what had happened. I asked her, “what should we do?” She told me I was pregnant. I asked her, nurse what am I going to do? How will I face my dad? Then she told me you are still schooling so we terminate this pregnancy. Then I replied to her “if I happen to terminate this pregnancy, doesn’t it equate to killing a human being?” she told me you are schooling how are you going to tell your dad that you are pregnant they will even chase you away from home and you are still young. She told me you will have nowhere to go, do you know where that man resides? But we didn’t even know where he lives. I was feeling ashamed… I wasn’t fat at that time, I was small but tall. I could not imagine people seeing me pregnant at 14 years, then they say that girl misbehaved at that age.

How about your father?

Even dad didn’t know where he resides. She said you don’t know where this man lives and then you want to face your dad and tell him about the pregnancy? Actually, I even didn’t have his phone number I even don’t where he is right now. She told me we are going to end this pregnancy.

So, she said, “I am going to give you this tablet when you reach home insert it in your private parts that pregnancy will be terminated. I replied okay nurse. I went home and inserted it, whether I inserted it in a wrong position I don’t know, I started bleeding, then I went back to the nurse and told her bleeding didn’t last long so I am not sure whether termination is complete.

Then she said let me check you and I accepted. She checked me and said that it didn’t come out, that it moved to the fallopian tube. I asked her fallopian tubes? She answered yes and right now I have to immediately run to the hospital. I asked her where should we go? That you come tomorrow and we go to Mulago hospital the following day was weekend.

She gave you one tablet.

It was only one. She told me you come and we go to the general hospital the next day. So, I waited for dad to go away for work. He used to ride a bodaboda and I also went to school, when I arrived we boarded a taxi with the nurse and went to hospital. When we reached there the nurse talked to the health worker but at that time I was feeling abdominal pain, I had pain in the lower abdomen. She told me you lie down here on the bed so that I can go and call a doctor.

The doctor came and said I need to be operated. I asked the nurse do you mean there is no other process apart from the operation, like giving drugs or anything? Then the doctor said let us see. The nurse told me now things have become serious let’s call your father, I asked her, what are you going to explain, to him. She said don’t mind I will talk to him.

Then she called him. I don’t know what she talked with him. She moved outside and called him, dad came. She didn’t tell me whether he learnt about it, dad came and asked me, “are you sick?” let me go home and bring some items we can use. He rode his bodaboda and left. I remained in the hospital with the nurse.

Who provided transport fee to the hospital?

But it was weekend and no schooling I have to remain home. As dad was riding back to collect some items along Queen’s way he got an accident and knocked his head on the pavement then he was rushed to Mulago on reaching there he was dead. That there was a drop of blood in the brain. Mummy came but she didn’t see me. So the body was collected first was taken home and later transported to here-in the village. The nurse told me they should attend to you before you leave.

Before attending to you, you learnt your dad had been knocked?

No, I wasn’t aware. But in that process, I was given chloroform, when termination was complete I was left with a cannula that I should go back every day to get drugs. On reaching home that’s when I was told your father is dead. We stayed there crying and then came here in the village on that night.

We were renting where we used to live and burial was to take place the following day. After community members looking at his body we were transported to the village using those funeral service vehicles.

When my mother saw me with a cannula, she asked are you sick said I have been sick suffering from typhoid, I came along with drugs provided to me and the nurse came for burial. I remember it was a Wednesday after burial she left and returned to her home after giving me the required drugs.

Any drugs used through the cannula?

I just remained with it because I feared to remove it thinking I might harm myself and bleeding occurs. She prescribed to me the drugs. I remained and stayed in the village for one week. But the nurse kept on calling how is she? I could talk to her and inform her I am feeling well, until we also went back.

While in the hospital what was the process you went through to end your pregnancy to be told to get up that termination is complete?

I don’t know, you know by that time I was given chloroform , it was given through an injection on the hand. I was still conscious but after administering it I felt dizzy and that was the end.

Just here (at the shoulder). When I woke up the nurse told me ‘everything is finished get up, we have to rush and leave this place because it is getting late.’ She never told me that my father had passed on. ‘We should hurry and get home your dad is about to return home’, I asked her “didn’t he return to bring items required?” She said no, you hurry up and go home. On reaching home I could see many people there, I asked her, nurse what has happened? Then she told me let’s first reach and find out. When I reached people were telling me that your father has died. I found there mum, all aunties from Kampala and at around 9:00p.m we set off to come here in the village.

1. TRANSCRIPT NAME: *Participant 2_Kampala, age 42*

Madam I would like to know the word that is used in this community to mean abortion

The word that we use to mean abortion, some of us use “ kujjamu lubuto” . most people say that “gundi yajjemu olubuto”, for those at school use abortion. They say that “gundi yakozze abortion” but most of us say “gundi yagyemu olubuto”.

What do you mean when you say that “for those at school”?

For people at the school for example where I am working, most of them use English so they say that “so and so aborted”

Is there any other word you use to mean abortion?

No, those are the commonly used

Now, using your estimation, can you say that abortion is common in your friends and family members?

On one side, abortion is common though not in my family members. Most of them…. i say no

Which side is that you are referring to?

Most of them are my friends, as you are chatting one can say that I conceived but I did this and this, you also say that I had it but because of what happened to me I did this and that

Why is it more common in friends than your family members?

At the moment I do not reside where my family members are. I have been in here for quite a number of years. At the moment there are some people who are more like my siblings. I take long without getting personal information about my family members. Ok they can call to say that this one had a miscarriage but not abortion. Apart from me, the rest are married. We are like 3 girls

What kind of friends do abort?

Okay, we have banyankore’s, bagandas and like 3 bagishu who aborted and some of them I did accompany them because if someone says…... and if this person is your friend and she says that I have got an issue but what should I do? They request you to accompany them, things of that kind

What kind of work do these friends mostly do and what is their marital status?

Most of them are single, those that aborted while still in their marriages also got issues with their partners and separated, they are now single. One mugishu got misunderstandings with her husband and they parted ways. He was not providing for the family yet the youngest kid she had by then was very young so she decided to go and abort

Which work do they do?

Some of them work in restaurants. You cannot work in a hotel when you are pregnant… some work in bars. Their income is daily which is just for food *(survival*), the person responsible for the pregnancy will not support her, she will think through it and she will say that let me abort because when you abort it will take you like one or two days to stabilize though with some mild back pains. If it you have no issue, you can abort today and resume work the following day. However, most restaurant tasks require bending so the blood flow is heavy. When you work immediately after aborting you over bleed

So most of your friends work in restaurants….

Yeah, restaurants, bars…things of that kind. They are for survival…stalls …

Focusing on your friends and family members, how do you get to know that there has been an abortion?

Its only when one has told you, you can’t just get that information…..a pregnancy of 2 months is just blood, at 4 or 3 months is when one can know that you are pregnant but 1 or 2 it’s the lady herself to tell you that I have missed my periods that is when you get to know that she is pregnant or sleeping all the time, cravings that is when you can know that she is pregnant. There is someone telling you that I am pregnant but I am going to abort, others you can know that she is pregnant but after sometime you don’t see her with the pregnancy you just know she aborted and especially if she has ever told you that she is going to abort

What else can notify you of a pregnancy termination?

If they are close you can easily know but if they are far it is hard

You said that most of those that aborted are your friends, how did you know that they aborted besides those you accompanied?

Some call and tell you or come physically to tell you that things have failed. Like I have a friend who is my best friend, she came and physically told me that I am pregnant but things have failed. She tried to also pull ropes with the partner for support but the man refused. She was renting her house and she in a restaurant. You can’t work in a restaurant when pregnant, you say that let me at least go and abort. You can know only when one has confessed that she is going to abort

Do you also hear from other people? Someone coming to you and she tells you that so and so aborted

Yeah, also those happen. Especially if those who have aborted are not your friends, they come and tell you the other one has been pregnant but she aborted. You know women with rumormongering

You said that some come and inform you when they are going to terminate, why do you think they confide in you?

It’s not because it’s my hobby but if you have been friends with someone, you have never quarreled, nothing bad has happened between you, you always visit her, that is why I get to know. You can advise her not to abort, you tell her to first have a discussion with the owner of the pregnancy, she says that how will I survive in this state, I am renting and I am a single mother, such things lead to that. But if your partner gives support even if you are not living together, you can give birth. What makes us to do that are the conditions

What else?

Because I don’t release secretes. I have only done so to you because you came to learn about us. They don’t confide in me because I take them to the doctors to abort, not that. There are some people you tell something and they don’t release it. There is also one telling you that I am pregnant and I run to tell this one, that one…. There are friends among friends that you can trust, you say that let me tell her this and this. You don’t have to tell all your friends no, it isn’t like that

Could it also be because they are aware that you also aborted?

To some, it’s that. As you are chatting you can tell them that I did it and I reacted like this. Some tell you to get advice. Some want to see whether you can stop them yet they have already made up their mind because of what they go through

How about them telling you because you live close to each other?

Its not like that. Not all of them are close. You can have a friend who stays in Kawempe. The gishu lady I have told you about stays in Nabweru. She called me and I went to her place. I advised her to hold on a bit as we wait for feedback from the partner. She said that the man refused. I leaving my marriage it was not a one-minute discission, you first struggle and struggle then later you leave if things fail

Okay. Now, I would like you to tell me about what women in this community talk to each other about?

About pregnancy or just a discussion?

Anything. They can be health related or any other topic

They discuss about family planning. They say that if things fail go for family planning. Things of that kind. Here we have kawaala and kawempe but kawaala is the most convenient to us. They say that when you go for family planning use injectables or IUD, things like that

What else do they usually talk about?

Testing for HIV to know our status. We have health workers who moved in this community drawing blood but there are tablets they gave out…. that those tablets prevent HIV transmission. I asked them about my status, they said that it is good. I then asked them whether I should go a head and swallow the tabs, I still have them kept in the house. But I went and inquired from one of the doctors around, I asked him that “won’t I get side effects if I take these tabs”? He said no, you should not take them because you have no man. They gave them out and said we take one per day for 30 days and this was to be done every month. I have them at home I will show you after here. I go for HIV test every after 3 months

What else do you discuss? They don’t have to necessarily have to health related, it can be a different topic

Being a single mother, I don’t involve myself so much in conversations for marrieds but when we meet as women, women usually discuss about men, that a man did this to me, he got another woman such things. That is what we mostly discuss. Topics about men. That this one fell in love with this one, another says that I have got a new boyfriend or I am calling my boyfriend but he is not picking up, such things. This is especially when the unmarried gather. If they are married, they say that “mine came back at 11pm, another says that “mine didn’t leave me with “kameza” (*upkeep*). Those are women chats nothing else. Mine is called at night when we are sleeping, mine left very early in the morning, we quarreled and that stemmed from the late-night calls. Such things

Okay. Don’t they discuss things to do with jobs?

We do discuss jobs, one time we invited someone who promised to boost us in that areas. We wanted to start up businesses…. like me, I know how to farm. I said that if someone supports me with money i can start rearing chicken. I don’t remember that MP’s name but he made us to gather under that tree promising to give us starting capital to open up charcoal stalls…. he used to discuss issues to do with developing women so that we also work and save money in our circle. We had started making briquettes but all those failed. We lacked someone to support us. However, some women here rear chicken, some have stalls, some make briquettes though we have no outside support

Ooh okay. Now, are there certain topics you don’t talk about with your family members or friends that are close to you?

Conversations I cannot discuss with my family members but I can with my friends …. like that pregnancy, I did not tell any of my family members including my mother, I feared to be ashamed. I had a kid already who is about 10 years now, I again conceived, I asked myself that what will I tell them? I came to look for money and there I was, pregnant…... I never told them. It is easy to confide in a friend than a family member. There are some sisters who are not friendly, you can have a friend who is like a sister to you and a best friend. It can be easy for me to confide in that one

Which topics can’t you share with your close friends?

What I can’t tell my close friends…. okay it is revealing things concerning my family for example telling them how my sisters are doing, those are family issues, and issues about friends should always remain in our circles as friends, you get? The best friend I have been telling you about knows our home and my siblings but still I can’t tell her that do you see that sister of mine, she did this and that to me, no there are things that are supposed to be kept to yourself. And with us single mothers, if you get a boyfriend, you can’t keep on opening up to everyone that I got this one, it is shameful. If you tell them about a boyfriend let it be that one boyfriend that you keep referring to but not all the boyfriends you get. And it isn’t good for your friend to get close to your boyfriend. If your friend is loose, she can take over your boyfriend especially if your friend beats you in certain things for example you maybe slender yet your friend is fat with big bums, you maybe very fat and the other is small with an outstanding figure, the guy can easily shift his attention to your friend.

Are there other topics you don’t share with your close friends or your family members?

Uhmmm, no

Is there any health-related thing that you can’t share with your close friends or family members?

Uhmm, sometime back I think it is now 5 years ago, before I started testing, the father to my kid…there is a time they used to say that he womanizes, they told me that he was in love with an infected woman and I started worrying. One time I got a boyfriend, he told me that we are going to test for HIV, I said okay, no worries but I trembled. I confided in a friend of mine. I told her that I got my person (boyfriend), I want to go and test but I am afraid, she comforted me and told me to regain my strength and go for that checkup. We went and when I arrived at the clinic my heart started pumping very fast, I said no…but it is because I had it in mind that my partner by then was HIV positive which I had not revealed to my friend. I went to where the nurses were to have my sample taken. After drawing the blood, I sat in one corner as I was waiting for the results but my friend was telling me one thing; be strong. When the results came back, I was having serious palpitations. The doctor told me to relax before revealing my results. He gave me some minutes to calm down and then told me that I was okay and advised me to maintain the status. But still that was not satisfactory to me. I went to another facility to test and confirm my status. They still gave me the same result. I then went into my relationship very happy. When we went to test with him the results were okay. From that time, any person who wants to be my partner, testing is a prerequisite. Without it I can’t be with him.

Okay. You said that you confide a lot in your friends…. you tell them your private or personal information. Is there any other person that you confide in?

With personal information it is my mother. I tell her that this and this has failed. There are some things I can’t tell my friends but share with my mother

Who else do you confide in?

I have a sister that follows me, I tell her when things have failed

Okay. If a woman has had a pregnancy termination, who can she tell about it?

It depends on where the woman is residing. For us the immigrants, we can’t travel back to our regions to tell your mother but share with your friends. If you are staying close to your family the first person you tell is your mother and that is if you relate well with her. One can be a mother but not a friend to you. Such people when you share with them, they mistreat or behave as if what you have done is a taboo. Yes, it is a taboo and not good but we don’t willingly do it, situations push us to do so. To us who migrated from our regions if you have a sibling in the district, you are in, that would be the first person to tell, but if you don’t trust her, or has no solution to your issue, do not tell her. Some friends are complicated, you can say let me tell her this and that and they disappoint. If you have a sister who is your friend and you usually communicate you can tell her just in case something bad happens to you. There is a possibility of having as serious adverse effects after termination, this sister can be of help to take you back to your region

Uhmm. Why would you confide in your close friends?

It’s because my family is not where I am. My friends live close to me that is why when I am sick, I call them to come and give a hand

You have said that you migrated to Kampala and most of the people close to you are your friends, but still, I know that you work at a certain school around where there are teachers and nurses or any other person. Why is it that you decide to confide in friends and not these other categories of people?

Take an example of that lady who has just passed….it is not that every person in a society deserves to be told your secretes. You choose someone to confide in. That lady who passed here is my friend and a workmate, I tell her to get advice since she is older than me. Different societies have different people with different characters. There are some who talk a lot and if you confide in such people any time you make a mistake, they can easily release your information. That is why I don’t share with anyone I find. I stop at greeting them

Hmmm. You mentioned earlier that there are some people you know who terminated pregnancies and you got to know because they wanted your assistance, another one confided in you because you had opened up to her about your termination so it was easy to trust you. What could be the other reason they confided in you?

First, she doesn’t come directly to inform you that she wants to abort, no. she first explains to you that I conceived but the man responsible is running away from his responsibility, he is not picking my calls, he is not taking care of me and yet we are living in a rental. I don’t decide for them to abort, no. it is from the bottom of heart that she makes a decision to abort. She asks herself that for how long will I carry this pregnancy yet I am renting, I earn daily and for food only, what will I tell the landlord…I have no reliable job. If the one who impregnated you loves you, you love him back, you have your own business, it is not good to abort that pregnancy. You never know the kid you are aborting can be a minister tomorrow, s/he can be the one to support you, maybe s/he is the only kid you have in the womb. It isn’t good to abort but circumstances force someone to do so

Do some tell you after termination?

There is one who told me after termination but she had the same story. A man impregnated her and this guy was not picking her calls yet she had no food in the house. That was the reason for aborting that pregnancy

You shared with me earlier that the last termination was in 2018. I would like you to share with me how you got to know that you were pregnant at that time

I was on family planning…it was 3 months injectable. At the time of sexual intercourse, I was sure that my injectable had not expired which was not true. It was an oversight. I missed my shot and that is the time I engaged in sex. When I reached home, I checked to see the dates I was supposed to go back for my shot, I found when it had expired. I took a pill after and left everything at that thinking it had worked for me. later I discovered that even the pill I took was expired. I thought I was going to have my periods but in vain. I waited to have my periods as it required by the health workers before going back for my shot but nothing was coming. I was shocked to see that I had gone into my 3^rd^ month without seeing my periods that is when I realized…...i had also started rejecting sugar, sweet things, matooke that is when I believed that I was pregnant…uhmm

Okay. Different diseases make people to reject things like sugar, matooke and so on. When those happened how did you confirm that were not sick but pregnant?

I am not that educated but I have wisdom, I went to a clinic and gave them 5,000ug shs, they took my urine sample…I can also get those things (strips) and do the check up from home but I said no let me go to a health worker to check so I can know the truth. After running the sample, she told me that I was pregnant. I also know when I am pregnant. If the red line is one, know that you are not pregnant. If they are two…. before she had communicated to me, I had already seen that I was pregnant. Maybe she even thought I was naïve about it but I had read the lines already.

Uhmm, so how did you feel?

*Before testing I was not feeling well and when I confirmed the feeling became worse. I said to myself that I have no job, I have no permanent place of residence. I came [to the city] to look for money, I have a school going child, I don’t know what to do. I tried calling him [partner] so that we could meet and talk,* he accepted. *When we met, he told me that “I am not interested in giving birth at the moment”. I asked him what I should. His response was; ‘you know better.’ I went back thinking that it was a joke or the response was out of fear. I called again, he never picked my calls, I called several times without any response. What would I have done? Someone was housing me with my child. I had just got my job. My next step was to abort. When I consulted my friends they also advised to abort. I didn’t intentionally do it and that is why I told you that if you have somewhere to stay, please settle and give birth to your children. It is not good to abort.* There is girl who was helped to abort. This is a friend of mine who helped her child to abort. They are not here in Kampala they are in Arua. They did it because the child was still school, unfortunately till today the girl has never conceived again, she has tried different men but in vain. That is the problem with aborting. I never conceived from my parents’ home but even then, I wouldn’t have terminated because that can be your only ova that God gave you. That is why I always advise my friends not to terminate

What process did you go through to make the decision to terminate your pregnancy?

*I was patient thinking that he would change his mind, I waited and waited but I was becoming weak. I could vomit everything I ate, getting out of the house was hard for me yet I had to work.*

What happened next?

I was patient but still I had no money. I told my friends about my condition and one of them lent me money. The doctor I went to didn’t ask for a lot of money he asked for 70,000 ug shs since the pregnancy was still at its early stage. Other doctors ask for 100,000 and above

Did anyone help you in making your final decision?

No. It was my own decision because when you are going to the procedure room you don’t go with any other person, they just supported me with money. My parents don’t know about it

Save what you already mentioned…. You said that your partner rejected the pregnancy, you had not a permanent job, you never had your own house and that someone was housing you, you were still caring for a kid that needed your attention and yet you never had money. Besides those, is there any other reason for having a pregnancy termination?

Uhmmm, I had no other reason. If he had given me support and also had a clear place of residence I wouldn’t have terminated because I loved him and he also loved me back. The pregnancy brought issue and what I know is; some men fear responsibilities, they want grown up kids and also feared the information to leak to his wife. There was no other reason

So, the man was married?

Yes. But I had no other reason for termination. Honestly by the time you accept to have sex with someone it means that there is something that connects them, which is love. It was not coercion or rape. If you willingly undress and he also undresses it shows love. He loves and you also love them back

Uhmm…okay. You said that when money was given to you, you went and saw a doctor. Can you please describe all the processes you took to end the pregnancy?

They were not so many. When I reached…. of course, when you tell health workers that you are pregnant and want to terminate, s/he says that okay, come we examine you, they examine to prove what you are telling them. The processes I went through were lying on the obstetric bed, opening the legs widely, the nurse came and injected me. later she inserted a tab which is not known to me and asked me to put my legs together because they expected the bleeding to start anytime. The bleeding was not heavy. After that, the doctor came and washed me (dilated/cleared the uterine lining), he then gave me tablets to take on reaching home. I also got ‘kamunye’ (local herb) and used. It isn’t for abortion but can be used even after a successful delivery. It is good for cleansing the uterus

Hmmm. Is there any step you took before seeking care from that health worker?

No, I didn’t use any other but there is a friend of mine who lives in the village, when she conceived, they recommended some herbs but she nearly died. Some told her to insert cassava flowers in her vagina, taking boiled soda(*coke*) but she almost died and that is why I never opted for that

How did you find out about the clinic where you went to have the pregnancy terminated?

I have friends who have stayed long in this area, they are the ones who directed me and also accompanied me. I first surveyed to know the prices and also interact with them before my appointment. They told me the process. You don’t just wakeup to have a termination done on the same day. You have to go and tell them your issue, agree on the amount to be paid, after that you go and look for money. Termination doesn’t take long but this is to those who seek for medical care not coke procedure

What challenges did you face during the process of termination?

I was not challenged in anyway apart from worrying about what we were told when we were young. I remember they told us that in our family people do not abort and that if anyone tries to terminate, they would die during the process. That was my only worry that time. I dint rush to make that decision because of that and if my intension was to terminate, I would have done it at one month

You also said that money was a challenge too…

Yes, and ended up asking from my neighbors yet they were not responsible for the pregnancy

Uhmm..Did you have any termination method in mind at the time you went to consult from the health worker?

No. I left them to decide for me. The only method I knew was the coke method which I couldn’t use. I went to experts because I knew nothing

I would like to be sure of the money you said was used to pay for termination. You said that you paid 70,000, is this the only money you paid?

I paid 70,000 for termination, I was then told to buy tablets at 25,000

So, all together it costed you 95,000 ug shs

Yes

Okay. Is there any non-cash item that was used to pay for the service?

No

Ok. Is there anything that the health worker did or said that was challenging to you?

No. however he only inquired whether I had ever used family planning. I told her that before conception, I was on family and that was not intended. She further inquired whether the decision I had made was joint or if we had had a discussion as a couple. I told her that I informed him but his response was demotivating. She finally asked me for my decision. I told her that I have decided to terminate it.

Okay. I would like to give you an example before asking you this question. When we want our children to start schooling, we first have a survey on the performance, security, hygiene of the different schools before making a selection. Now focusing on health facilities, what qualities did you consider when choosing where to go?

I didn’t have any qualities but *What enticed me was the availability of a doctor, very professional and he also works in known hospitals. I selected it because when things fail at his facility, he could easily refer me to the next level. I also went there for privacy, I wanted to be treated from a health facility and come out well without my partner at home knowing what had transpired. All I wanted was to be in the hands of a trained personnel…*

Hmmm. Were costs one of the things you considered?

*I went to two clinics; the first one was asking for 100,000shs, the second one my friend who had accompanied me was known there, so I told them that I didn’t have money and they reduced the cost.*

How did/do you feel about the way they treated you when you went to have your pregnancy termination?

The health workers treated me well, they comforted me and gave me water to bathe. I padded myself after bathing, after that I went and sat somewhere. I had mild pain but I was given tablets to swallow. When I reached home, I went and rested

Did you receive any information from the health workers at that time?

It was only about family planning and nothing much was said about abortion since what they prioritize is money. If someone knows his work all they have to do is to welcome anyone who has come with money

Did you have any question for the health workers or did you ask them any question?

I asked them about the solution for my pregnancy since it was still at an early stage. They told me that they knew what they would use, and then asked me to go to the procedure room where the process was done from

Okay. You said that they injected you, inserted a tab, dilated and also gave you tablets to take. Did you feel that it was a safe way to terminate a pregnancy? You talked of people who used unsafe methods and nearly lost their lives. Do you think the method was safe for you?

Yes, because comparing it to what my friend used, I feel it was safe

Uhmm. You shared with me your experience while in the procedure room, what they did and how you felt. So, what happened to you when you reached home

I was somehow weak and also having that feeling that what you are from doing is a taboo although I had nothing to do. If my mother had aborted me, I wouldn’t be in this world. All that happen because of the person who impregnated you. If these people were taking on their responsibilities, we wouldn’t be aborting

Is there any other thing that happened to you physically/physical discomfort?

I was not that sick, I only felt pain in the lower abdomen but I continued with my medication

How about the bleeding. Can you please describe how it was after termination?

It was not that heavy. It was intermittent after termination

Did you experience any symptom that made you to feel like your life was in danger?

No. it didn’t actually take me many days to resume work

You said that you felt some pain in the lower abdomen, did you seek for treatment?

The medical form I had had his number so I called him and he told me to continue taking the tablets I was given and requested me to go for review. When I finished my dose, I never had any other issue, I never went for review. And with this situation, I didn’t risk going back to the clinic. Review required money so when I normalized, I didn’t bother going back for review

Okay. How did you know that your pregnancy termination was complete?

When bleeding started the doctor came with something similar to a trough (*doctor’s tray*) which was having equipments. I then saw him inserting an equipment, I surely don’t know what he did but he told me to close my legs when the procedure was done. That is when I realized that it was complete. I asked him that doctors are you through, he said that yes, you are now okay.

*(respondent looked exhausted). We are about to finish.*

*(I just have pain around my neck, I fell but since then I have not fully recovered)*

*Sorry, let me summarize….* Is there any thing you would like to share with me about the treatment you got from your friends, health workers or any family friend?

They treated me well but my problem was how I reacted to that pregnancy. I weakened, lost appetite for food so if i start developing such signs, I just confirm that I am pregnant. I never encountered any negative attitude towards my decision but my only fear was what my family members said about those who abort. That was my greatest worry. It was sole decision and none of my family members know about it. My life went back to normal…I never bothered calling the other one (*the man who was responsible for her pregnancy*) again and he also never bothered looking for me. I was challenged and I swear never to get pregnant again

Why don’t you want to conceive?

I don’t want (sighed)..

How many children do you have?

4, the 5^th^ one died

Only 4 children and you are thinking of stopping!

It is mainly because of the situation. I have no stable job and no business at the moment yet I really desire to do farming. If I have capital, I can go to my village and plant rice… this is a season for rice growing.

Did your pregnancy termination affect your relationship with your family members, friends or people you know?

No it didn’t affect them….i never told all my people, I did select a few and some of them were informed when termination was complete and after some good time as we were chatting. I let out a few things

The effect can be positive or negative

I was affected negatively and in that I was able to learn a lesson. These days I no longer rely on men, I have to take care of my children as a single mother. I actually disconnected myself from men. I will do whatever job I find to see that my children go to school. Men come to me but I know they are all the same. Marriage is good when you get the right person.

TRANSCRIPT NAME:  *Participant 4_Rakai, age 28*

When did you make the final decision that no I shouldn’t continue with this pregnancy? What did you consider?

Depending on the partner’s actions; he wasn’t bothered whether i existed or not, he could no longer pick my calls, then I thought if he has started to behave like this, what will happen if I give birth? He was no longer supporting me. He picked me from your home and brought me to a place I don’t know…There is no one I know. I was very far amongst the cattle keepers, whereby it was very hard to find a Muganda you can confide in, they were mixed tribes in Kakuuto, they are cattle keepers.

Who made the final decision?

It was mine, I had made my decision that I have to terminate this pregnancy.

Let us assume you continued to be supported what could be your decision?

Don’t make jokes...

We are just assuming that care and support were being provided, and he could also be seen around, would you still have this decision?

It would not come at all, in case I was getting all what I wanted, you can take heart and give birth to your child because you can be sure that since he has been able to take care of me during pregnancy then he is capable of catering for the child. There was totally nothing, no, that is when I made up my decision.

Did you inform this person what you were going to do?

This man, I informed him over the phone that I am going to terminate this pregnancy, and he told me that, “the moment you do so, we don’t carry out abortions that means you will die”, then I told him no problem let me die.

After telling him, plus your friend did you inform any other person about what you were planning to do?

No, no.

How about your family members?

No, none of them is aware that I have ever done it.

How about the health worker?

Maybe the health worker who attended to me, plus the other one who provided treatment to me the second time.

When did you visit this first health worker to inform him about your plans?

*This friend of mine was already a friend to him [health worker]. I wonder whether she had aborted before. She was taking there many people. The health worker was well known for conducting abortions, they always have their phone numbers. Actually, she just called and told him I would like to bring my sister. T*hen he said “you bring her”.

Did your friend help you in making your decision to end that pregnancy?

I personally told her about it then she said that, “should I direct you?” *So, she directed me and escorted me too*

Apart from this gentleman not supporting you, did you have any other reason why you didn’t continue with this pregnancy?

He already had his wife, I was still a young person so at my age, how could I join a marriage with another wife plus children? To me I felt it will not be wise to do so by then, but right now it is possible.

Today you cannot do it?

Yes, by then I was still young and at the same time he was old, but right now I can get married to a man who has his wife if he gives you somewhere to stay, if he builds a house for you it is possible, but at that time no.

Was this the best preferred place where you went to be attended to?

I *wasn’t aware of any other place. I didn’t know anywhere* and it was my friend who directed me to this one. I didn’t know anywhere.

I understand, but when you reached there, did he have the necessary equipment?

For me I was after removing it so I didn’t know about that, whether he had them or not, that was my target. Besides I didn’t even know what they use, no, I didn’t know.

What challenges did you face during that time that were hindering you from meeting your goal?

Challenges I got during pregnancy termination?

Yes, at that time what was difficult for you?

Money, at first it was hard to raise the money, I had failed to get money required, he had demanded 150,000/- and he said less than that let her not come, so I looked for it in good and bad faith (n’engwa ewabi n’ewalungi), I looked for money and we got it, then we went.

It was a money problem.

Yes, it was money.

What challenges did you face again during the pregnancy termination?

I bled severely and in my life I realized a person can bleed to death, by then I didn’t know, that is when I realised it would happen.

During that time, you had severe bleeding as a challenge, what other challenges did you face?

Nothing, it was the bleeding which affected me so much up to the extent of weakening me.

How about the health worker, did he do anything which made it difficult for you apart from money?

No, because when I got to her and I explained my problem, she attended to me promptly. When we went to the room and I told her my problem she didn’t hesitate she just attended to me. She worked on me quickly and actually she gave me care.

The first health worker?

No, the second one.

No, I am talking about the first one.

I said that health worker when he finished there were certain tablets he gave me to take and I thought it was already done.

So, he didn’t do anything difficult for you, this first health worker?

That one as I said he really delayed us, he was busy with his patients, yet for us we wanted to go back.

How about when you visited the other health worker to explain to her what you were going through, did you get any problems?

No, I was attended to very quickly because I told her I carried out a pregnancy termination but this is what I am experiencing, she gave me care immediately, she gave me IV bottles, she surely gave me treatment surely.

Didn’t he demand for money before treating you?

No, private facilities don’t demand for money initially but they ask for it at the end, unlike these government facilities which ask for money to buy cannulas.

How about your friend, did she make anything difficult for you?

No, my friend really cared about me, I think she realized that I am in a bad condition, she was worried that ‘people saw me taking you’, because people are watching, so and so went with so and so, she really cared for me, indeed she did care for me.

Finding a place where you could go to be attended to, was it a problem to you?

No, she knew the place very well.

Now you paid this first health worker for the pregnancy termination?

150,000/=

How about the second one?

The second one didn’t take a lot of money, I had that money it was my salary and it was my friend who told the boss that I had fever, my friend is in the clinic she has fever, so the lady just got money and gave her, I had worked for a month so they came and paid the facility bill.

How much was it?

I remember it was less than 60,000/=

That is the second health worker?

Yes, it didn’t add up to 60,000/=

1. TRANSCRIPT NAME: *Participant 11_Rakai, age 25*

How did you find out that the health worker was the right person who could help you with that challenge?

She has been a longtime friend and first when I told her she said no, go and see some other health workers, so she directed me to go to Dr. Matovu because he can help you with that pregnancy termination so when I went there he told me to bring 200,000/= which I didn’t have. I went to another place and the other health worker too told me to bring 200,000/= and honestly I didn’t have money. So when I came back and told her … she didn’t want me to abort and she was so touched, she was so troubled about it but when my husband arrested me and ashamed me everywhere, he said “the pregnancy is not mine”, among tenants, my dear we were exposed before the whole world [everyone]. He said, “once I get tired of someone then I mean it, if the pregnancy was mine I would have helped you and taken care of you but that one is not mine.” The man talked too much.

So this health worker was my longtime friend, it’s not this incident that brought us together but she was my friend to an extent that I can go and tell her about different issues. But they were tribe mates with my husband, actually he connected me to her when we were still in love, I would share with her everything that happened to me like your brother has done this and that to me. That’s how she ended up becoming my friend and she is a kind of person I could tell my problems that this is like this and that. So whenever we had an argument she could ask me for my husband’s phone number and she could call him, at some point he no longer liked her so he couldn’t pick her calls anymore.

This is because he knew that she is calling him to talk about our relationship yet he was not interested any more. He said, “That woman is still young why doesn’t she get rid of me and get other men, you know that I have a wife, but this one is clinging on to me because you will call me when my wife is listening.” So when I explained everything to her, she said, “you would have left that pregnancy and produce your child.” Then when time reached, she listened to what the man was saying that’s when she said if you are determined to terminate it, please do it.

When she realized that it is too much

Yes.

You said you knew the other place already.

In their place?

No the traditional herbalist’s home.

Okay that one [traditional healer] was my friend because she knew us from childhood. I could hear about her as an old woman who usually gives out herbal medicine. So, I thought that because she is a herbalist, she might be knowing these medicines to use. That is why I went to her. I deceived her that it was my friend who needed the herbs.

Is there anything that the health worker did and you felt that it made it difficult for you to have the pregnancy termination?

There isn’t anything because I am the one who decided to terminate it because she didn’t want me to do it but according to the way my husband was treating me I think she felt touched as a woman and then she told me that if you are determined because she realized that I was determined and I told her that come what may I am going to terminate it. At least let me die with it and leave the world other than suffering. She told me the medicines but she didn’t just give me as a friend, I had to pay.

Only that what she helped you with was to give it to you in bits whereby you had to pay later on.

But I had to pay for the very first medicine, these other things such as medicines, cotton, gauze, which she continued to give me I paid later on.

You didn’t encounter any challenge of getting this place because you already knew it.

No.

And you didn’t inform any of your friends apart from the other one who was going to abort?

I have not apart from that one that was going to abort yet for me I had already aborted mine and her baby is now about two months.

About how much did it cost you in general?

I first paid 60,000/= and these tablets and the cotton plus gauze where she is demanding me 150,000/= which makes a total of 210,000/=.

Plus the other amount of money which you were supposed to pay to the traditional birth attendant, did you pay her?

I have not yet paid her (laughter for both)

So your friend has not yet refunded your money.

I think she even gave up, that woman of God.

Will you take her money to her?

I had even forgotten about it but sometimes if I have passed by and I have some money, I can buy some sugar and take it to her. It seems she gave up.

So ever since you got that problem you have never gone back?

Remember I had been chased away from home, I had not gone there yet but I went there of recent it’s not a long time.

When you reached there what did your mother tell you?

My mother is the one who called me on the phone and told me that “I forgave you because you got to know your mistake, come back home, I have no problem with you.” So when I reached there, as you know a parent, she took care of me because I wasn’t looking good a result of what I had experienced, even up to now I am not yet any better because I feel dizzy since I lost too much blood, I always feel too much headache, up to now it still happens. So I usually tell my friend Nurse and request her to give me tablets for headache. Then she tells me that you bled so heavily the body has not yet regained so as to normalize health wise.

So whenever you sat on a bucket how much blood did you lose? Like how many cups?

It would flow with this sound shuuuu and I would wonder what was coming out. The other woman had struggled to give me medicines to reduce the bleeding but things had failed.

What are the most important qualities in a place and method that you considered when choosing where to go and which method to use and the qualities of the health facility?

At first I had been told me that Matovu is the best Doctor when it comes to terminating pregnancies. Then I thought of going there but I lacked the money.

Wasn’t there a place of your choice, it’s like when you select a school and say I want my child I study from this type of school because they have a school van and the rest.

I had wanted to go to Matovu’s clinic but earlier on when I was still staying with my husband and while pregnant, he knew that I would go to Grace’s facility and that’s where I should produce my baby from.

Is Grace’s facility a private health facility?

Yes.

Why would you like to deliver from there?

This is because whenever you deliver from such a health facility, they are always available in case of any complications and at whatever time you need them since I had had a miscarriage I knew that I might get some complications in that if at all I go to the private health facility everything is available, they care.

How about at a government health facility?

Those ones don’t have care, by the time you call a health worker they are doing their own things, but here you are sure that the health worker will be available at all times you need and you can tell her whatever you want to be given. Yet in a government facility when you call them they abuse you, do you think we are attending to you only? “totutijjirako*” [literally meaning that she is assuming that she is the only person in need of health care and is exaggerating the condition]* shall we work on you alone?

How do you feel about the way you were treated when you went to have the pregnancy termination, the procedure or purchase the pregnancy termination medication?

She had no problem because she could even come and wash my clothes.

Did she give you any massage or any kind of counseling for example telling you that drink enough water?

Yes she used to give me counseling and she told me that if I feel any pain in my body, I should go and tell her and she told me to take care of my health because I started the process intentionally, no one forced me. Whenever I felt like I am discouraged, she told me to be strong. She counseled me before I terminated the pregnancy and she told me that abortion is bad some people die, some get complications. She gave me enough counseling before but my heart was already determined to do it.

I told her to give me tablets so that I can swallow them, if it means dying, let me die. When she wanted to give me that medicine she counseled me and didn’t neglect me. Every time I was in the house wondering in severe pain, she could tell me to smear your body with some Vaseline such that I don’t look pale. “You will look bad.” Whenever she got something which they have told her that it increases energy in the body such as glucose, she could but it for me and tell me to drink such that I can have energy in the body. She supported me so much. There are somethings which she could buy and tell me you are not going to pay me for this. She could bring a book and pen and we write. I could tell her that it’s okay whenever she could get fruits such as pineapples or passion fruits she could buy them out of her pocket and bring for me.

Did you have any questions that you wanted to ask her but failed to ask her?

No, I didn’t have them because whichever questions I wanted to ask her I could ask her and she would respond to them.

So that’s the kind of treatment that you used because you said she gave you tablets plus the traditional herbs.

Exactly but I didn’t tell her about the traditional herbs I used because she didn’t like it. So I didn’t. So I didn’t tell her about it, it remained on my heart and if at all I had told her that I used herbs, I would have discouraged her. The only thing she told me was that I should use banana leaves which are hot to massage my abdomen. Whenever she secured sometime as you know she works at the hospital, if at all she came back early, she could hurry and come and boil for me some water. She told me that let her first massage you and also do other things.

1. TRANSCRIPT NAME: *Participant 8_Kampala, age 33*

How did you get to know about their pregnancy termination?

She came to my home and shared with me what she was going through. I also saw that she was not okay and I asked her about it, and she told me why. Eventually she terminated the pregnancy.

So that one came to you, what about the others?

The others, you just hear when someone is gossiping about someone that so and so aborted, so and so terminated her pregnancy things like that. That’s how I get to know for some of those women who don’t share with me. I have one woman who shared with me and also asked me how to do it, and she went and aborted. Another one shared with me after the abortion.

So some share with you and some from gossip,

Yes

Okay, so moving on, in the previous survey, you said that you had done something to intentionally end a pregnancy. I would like to ask you some more questions about that. You told us in the survey, that your most recent pregnancy termination was in the previous year

Yes

Can you describe how you found out that you were pregnant at that time?

I realized that I had missed my periods after one month. I also saw that I had developed nausea, lost appetite and I knew immediately that I was pregnant. To be sure, I went to the health facility and bought a pregnancy urine test. They taught me how to read it and I understood it.

Was it a government health center, or a pharmacy?

I went to a pharmacy and I shared with them that I was not fine and wanted a pregnancy test. They offered to test me but I opted to do it myself from home. They taught me that if it is one line, there is no pregnancy, if there are two lines, there is a pregnancy. I went home, tested, and saw that there were two lines.

So you confirmed that you were pregnant?

Yes.

Okay, how did you feel when you found out you were pregnant?

I felt bad.

Why did you feel bad?

I was not prepared to give birth; the pregnancy was unexpected. However, I had a thought that maybe I should give birth to the baby but when I shared with my husband, he refused.

What was the process you went through to make the decision to end your pregnancy?

After confirming that I was pregnant with the test, I told my husband about it.

How old was the pregnancy?

It was a one month old. So I shared with my husband

Did you call him on phone or in person?

He came home and I shared with him that I was pregnant, He said, “ehh you are pregnant in this period?”

Why was he specific to that period?

I don’t know what he meant. He just told me ‘you are pregnant in this time? No’. So I asked him what the solution was and he told me to abort. I feared.

Was it your first time to abort?

No I had ever terminated a pregnancy before, but that was in the past.

It hurt me and I felt I never wanted to repeat that again. We disagreed about it a lot. I raised the issue of death to him and I asked him, ‘what if I lose my life? What if I am arrested’? But he insisted that I had to terminate the pregnancy. He insisted upon it until I aborted.

How long did this back and forth argument take?

We argued about it for a week and then I accepted to abort.

So whose final decision was it?

It was mine.

Did you have anyone else to talk to about what you were going through?

I shared with my friend. I called her and told her that I was pregnant and my husband had refused me to keep the pregnancy. She advised me that if he had insisted then I should do it as opposed to arguing with him continuously. Based on her advice, I decided to abort.

So it was your friend who eventually convinced you?

Yes.

What were your reasons for having a pregnancy termination?

First of all, the one responsible for the pregnancy had showed me that he does not need it. So I asked myself, should I really carry this pregnancy and then suffer with the child?

Why do you think your husband insisted that you should abort?

He insisted because we are two wives being Muslim. He fears his first wife so much, and so I think during that time he didn’t want her to know.

How many children does he have in the first wife?

He has one child with her.

And how many does he have with you?

He has two children with me. So I think he based on that, because he had not told her about me and he had hidden that away from his first wife. I think she had started to suspect that I existed.

How did the first wife find out about you? Are you in close proximity?

No, but I know she can be able to see me. Another issue he raised was his income. I hadn’t yet started working that year.

What work was your husband doing?

He is a health worker.

Traditional or modern?

*(laughs)* Modern!

So if it wasn’t for your husband, you wanted to have this child, right?

Yes. Mostly because I feared the consequences of abortion.

What else was happening in your life at that time?

I was so worried, I had no peace because we had failed to come to a conclusion.

What steps did you take to end your pregnancy? What are all the things you tried or places you went to have the pregnancy termination?

How long did it take after you told him?

It was a week

What happened next?

I then went to the hospital

Did he accompany you?

{Laughs} no he did not. He just gave me money. I went to the health center. I refused surgical procedures because it is what I used for my first abortion and it was so painful. This time they explained to me that there was a method that was not painful. He [male partner] had also told me about it. Eventually it came out.

What method was that?

They used pills, there are tablets that they inserted in me and the rest I put them under the tongue. I went back home. They told me go back home and sleep. It will come out. I thought that I would just feel it coming out but it was so painful

So your husband had also told you about these pills?

Yes, he told me there are pills they use, pills they insert. That if I go to the health center, they will put them under my tongue and also insert them.

Is that the only method you used at that time?

Yes.

Do you remember the type of pills they were or their names?

No, I don’t remember the names.

What kind of a health center did you go to, was it a government or private facility?

It was a private health facility.

How did you find out about these places where you went or the methods you used?

My husband is the one who directed me there. *When I confirmed to him [partner] that I agreed to abort, I asked him where I should go. He directed me to the health center.*

Was it easy for you to locate the place?

Yes it was. When I reached the health center, t*here is a health worker that he had connected me to. I went to him and he told me to lie on the bed and he inserted the pills.*

How much time did you spend at the health facility?

Not even an hour because when he told me that sleep there, I slept there and he inserted the pill. When he said move here, I moved. However, he was not so speedy, I think he had to target the entrance of the uterus, I never understood it well. He then told me to dress up, go home, and sleep. He told me I would just observe what would happen next.

Okay, what challenges did you face during the process of getting your pregnancy termination?

After the termination?

From when the process started, what challenges did you face?

There are no challenges I faced.

Is there anything that the provider did to make it difficult for you?

No, there was nothing.

Anything any friends or family did or said?

No.

Was there anything in your life that made having a pregnancy termination more difficult?

No

Was it a challenge to find a place or choose the right method for you?

No, I knew the method I wanted to use.

So they also did what you asked

They asked me, but I think he had finished talking to them. At home I had already told him that I don’t want to use the surgical procedures.

Did you pay for the pregnancy termination?

Yes.

How much did it cost?

I paid 100,000/-

Was paying for it a burden for you?

No, it was not a burden for me because the way I went, it’s my husband who had made the appointment for me, he had connected me to the health worker there. So it was not difficult because I knew what I was going to do.

Did he give you the money to pay or he sent it on mobile money?

He gave me the money to pay them, I moved with it

So how much did he give you verses how much you paid?

He gave me 100,000/- and the health workers also asked for 100,000/-.

What was the experience of your husband paying for you like? How did you feel about asking him to help you pay?

I didn’t feel bad, he already knew what to do and he had accepted it.

What are the most important qualities in a place and method that you considered when choosing where to go and which method to use?

In that health center, I found there very many people. I saw that it was a busy health center, and it looked responsible, I saw that the heath workers there looked professional. That gave me the courage.

How was the issue of privacy, was it a concern?

It Was okay. There was privacy.

Convenience?

Everything went well, I faced no challenges there.

How do you feel about the way you were treated when you went to have the pregnancy termination procedure or purchase the pregnancy termination medication?

I felt abit of fear because I didn’t know what would happen with the method I used.

Did anyone make you feel more comfortable, or less comfortable?

No; the health workers actually encouraged me that there was no effect I would get. I however remained with the fear I had come with because of what I had heard other people say. No one made me feel uncomfortable.

How did you feel about the information you received?

I felt stronger and that I was not as fearful as I had come.

So you came with a lot of fear, but the health workers’ words made you feel stronger

Yes.

How many health workers attended to you?

It was only one health worker, who gave me some counselling. He told me not to fear and that it wouldn’t be so painful.

What questions did you have for him and how did he answer you?

I had no questions for him.

So you just listened to him?

Yes, and I accepted whatever he said. *(laughs)* I totally trusted him. I felt okay about the way I was treated.

Did you feel like this was a safe way to end a pregnancy?

That’s how I felt.

Can you tell me why you felt that way?

The way the pills work, it’s as if you are just going to deliver. The way the machines work is also another way. So when I compared, the pills were so much better.

So what happened to you physically and after the pregnancy termination?

When I left the health center, I went home and straight away entered my bed.

What time did you go to bed?

I may not remember the exact time but I know that it was in the evening, like at 6:00 pm. I went straight to bed and I slept, slowly.

So when you entered in bed, did it start straight away?

No it did not start there and then. Sometime passed.

About how much time?

It was just a few minutes and then I felt pain in the tubes. It was hurting me. After some time, the pain increased, like when a woman is experiencing labor pains. I then went and sat on the bucket and it came out.

From the bed to the bucket?

Yes, I had prepared the bucket because the health worker had prepared me. He told me that within one hour I would start bleeding. Whenever I would feel pain and the urge to urinate, I would sit on the bucket. Until it got finished.

How long did you sit on the bucket?

I sat on it in intervals, I would sit on it and then go back to my bed. So when the pregnancy aborted, the pain decreased and I started padding myself.

Did you have the pads prepared?

Yes.

Were you prepared for the symptoms you experienced?

The bleeding did not scare me but what shocked me were the labor pains, they were so strong. I think the health workers opted not to tell me. All they told me after inserting the pills was that go and sleep, after sometime it will come out.

So they never told you to expect that level of pain?

No, they did not.

Can you describe the bleeding you experienced? What were your thoughts at that time?

In my mind, I had one thought, that I had really killed someone. That’s all that came to me, although I couldn’t see a person, just a lot of blood. I asked God to forgive me since I had overcome it.

And did you have any effects as a result of the bleeding?

I didn’t get any body weakness or headache, no.

Were you home alone at the time?

I had a girl I was this, that time but she would go early to work.

So did you feel any symptom that made you feel like you were having a health problem?

None at all.

Okay, so did you seek treatment for any of these physical changes that you experienced during the pregnancy termination?

He gave me some medication. What I remember, he gave me Panadol, and the medication that washes inside the womb, to press the blood inside the womb. Locally I also added on herbs like ‘kamunye’

At what point did you drink the herb ‘kamunye’?

I drunk it after the pregnancy termination.

Did you take the herbs on that very day?

No, I took them the next day.

How did you know about the ‘kamunye’?

I knew about it from women’s conversations. When you have given birth, to manage the inside of the uterus, modern medicine can work but also herbal medicine works. The pregnancy termination felt as if I was giving birth.

Who mixed the ‘kamunye’ for you?

I mixed it myself.

1. TRANSCRIPT NAME: *Participant 9_ Rakai, age 18*

Okay now we are going to do this, please tell me about your story, how was it? About getting pregnant and how you terminated it? Where did you go , please tell me.

We were at home where we stay; they were making repairs on the house because there was an upcoming function this year. So when they started the house repairs last year and I used to stay at home alone. My grandmother doesn’t stay around home during the day, i remain alone at home but builders were many but it wasn’t us cooking for them. We would just inform them that it’s time for lunch because we used to go and check at the restaurant if food is ready and then you go and tell their engineer .|Their engineer used to just be there to supervise them. I used to stay home but I used not to go where they were but as you know, they keep looking at you and times if you enter there, I do not want them to look at me. I would just be there and when food gets ready, I go and find out then I tell them that “they have said that you go for lunch”. So the engineer could always say “Flavia, Flavia”…

You used to stay at home alone

Yes, my parents used to come back in the evening so I used to remain there. The used to call Flavia, Flavia, they could call me, sending me but I didn’t want to get used to him because I used to treat him as an elderly person. When he comes i would go and kneel and greet him then he started saying that “You girl, I love you” but I used to tell him that “I will tell grandmother” i used to be there reading my books and because I couldn’t read from the front part of the house because motorcycles keep passing by and now here behind its enclosed and I would sometimes read from there inside a store. So he would come and say” why are you reading, you are not even going back to school”

Was that the builder?

Their engineer. He would say that “you are now adults and of marriage age, you are not going back to school, they are not going to open up schools now, if you do not have what to do , get married ” Everyone has what they want , if they want to get married they would not have endured for such a long time those who wanted to get married got married .

Now on a Saturday, they left when they had cemented but didn’t pour water there, so he called me and told me that “pour there water and remove even these things”. That is when he raped me.

Sorry. It was so unfortunate (Interruption).

So when that happened, whom did you tell?

I told him that I do not want to get pregnant then he told me that it cannot happen, then I told him that “You think I do not understand?” Then he said that it can’t happen. So when grandmother came back I didn’t tell her. I didn’t say anything for a whole month but when I missed my periods for that first month, I didn’t mind because I normally miss out my periods during some months but then the 2^nd^ month also elapsed and I felt changes occurring and during the 3^rd^ month I got a lady who was my very close friend whom I told.

During that time I used to call him but his phone was off, I even used to use the home phone to call him but it would be switched off and at times it would be busy. So there was a time I called using a different number and told him that “you black listed this number” and he told me that he has never blacklisted it but then I told him that” at times I call it and you make it busy.

That is when hen I told him that it seems I am pregnant”. Then he said that” no it can’t be “then I told him that “if you deny, there is someone I have told and if she tells my mother they may do something bad to you because you are an older person. Then he asked me, “What should we do?” I first told that lady about it so the lady said that if that man can send money, we would terminate it.

By that time I had also tried with tea leaves during the 2^nd^ month thinking that they can terminate a pregnancy. I used tea leaves but it didn’t get terminated but it failed to get terminated. One day that lady called and was tough on him that, “I can even imprison you, come and help some one’s child, you are the one who lied to her and yet you are an adult”. So we connected, met him and he gave us money, we went with him and terminated the pregnancy.

Where did you go?

We went to Kyotera in a clinic.

Okay so what did they do when you reached there?

I think he had already told the Musawo because when we reached there, my blood was tested and confirmed that I was pregnant. He first argued but then I told him that, “I told you, I told you.” *We went in and I was injected on my thigh and then terminated it [pregnancy]*

Were you conscious?

Yes

So what did they do?

*They just brought something like a tube and they inserted it there, you feel it inside here while outside here he is sucking / pulling it out. The pain was too much, it was so painful because I had never done it before.*

Were you sedated?

How?

Sedating so that you do not feel anything. There is an injection you receive…

I was injected here on the thigh and told that it would make me not to feel pain but I still felt the pain.

So what were you thinking about at that moment?

They had said that if you terminate a pregnancy, you die. So the man first went and spoke to the musawo and told him that I first do a pregnancy test but I didn’t go.

Why didn’t you go for the pregnancy test?

It’s not easy for you to just leave home because you will not be able to tell them that I am going for this.

So what did he say when you refused?

When I refused he called the other lady and also told her but I refused. She told me to go to that upper clinic that is near the hotel while they used to eat food but I told him that “I will not go there because I also fear being imprisoned also.

So what tricks did you use on the day you went? How did you leave?

At home?

Yes.

On the day we were going that lady told me to go to her and told them that there was a festival we were going to because I used to dance, maganda dance. So I told them that I had been hired and I was going and we indeed went. We didn’t delay because that man had already made a program so we came back early and told them that it was cancelled

Okay, so what means of transport did you use to go to Kyotera?

I went on a motorcycle.

How about coming back?

We stick retuned on a motorcycle.

Wasn’t the pain too much when you were returning?

No, after terminating it, I first felt dizzy and I wasn’t stable but he told me that you will be fine but when I came back, I had to be strong and secretive like nothing had happened because I didn’t want home people to know.

What do you think they would have done if they had known about it?

They would have forced me drop out of school.

They would have taken you out of school and yet you wanted to study?

Yes

Is there anyone who would have encountered the same problem and was forced to from school?

No but whenever she is counseling us she tells us that.

When counseling you?

Yes that if you get pregnant you dropped out of school because you will have gotten whatever you wanted.

Who says that, is it grandmother or your mother?

Yes its grandmother.

Grandmother always tells you that the moment you get pregnant you drop out of school.

Yes you drop out of school.

Now taking you back to the hospital where you went, when you reached there, did the Musawo tell you anything other than injecting you? You said that they first tested you blood to confirm if you were pregnant and injected you on the thigh, what else did he do? What other service did he give you?

Nothing else because when we reached, I think he had already spoken to him (musawo) so we paid money there to get a receipt and entered inside where he was ,then he checked me and said I am pregnant. Although I was carrying the pregnancy, I also did not know that I had it other than missing my periods was becoming too much but I had started feeling it.

How were you feeling?

I started feeling feverish, when I enter into the house, there was an odor that used to make me feel bad but it wouldn’t talk about it.

1. TRANSCRIPT NAME: *Participant 12_ Rakai, age 34*

How did you feel when you found out that you were pregnant?

Haaa!!! Musawo, it was a difficult moment…it was a difficult moment because I felt so so bad, Musawo I felt so so bad… honestly I cried for two days ((as she cries)) and whenever I think about it I feel so bad, it is a very big burden in my life. I went on overcoming it gradually, my friends too were comforting me… ((Interview paused, respondent in tears))

Okay… am sorry… but good enough it was terminated…let’s focus on the future now you will be fine.

I tried on my own as I thought of how to survive, how to take care of my children.

Where did you leave the children before going to Kiboga?

I left them with my niece but am now staying with them. When am stressed the children divert me a bit like when they play in a dangerous way and I have to shout at them then I stop stressing myself and I calm down. Whenever I look at them I gain courage because I must take care of them so I don’t think about the problem at hand so much.

When you went to the health worker to terminate the pregnancy what did he do exactly?

*I lay on the bed then he came in with some equipment on a tray. I didn’t see what they were. He told me to widen my legs then inserted some equipment and I felt as if he was pumping something.*

Were you sedated?

*No. I felt everything he was doing; the pain was too much. I held his arm. He told me ‘do not hold my arm, do you want me to spoil an organ here?’ Let me to do what am supposed to do.’ But I was in pain*

Didn’t he inject you before?

No he didn’t inject me. He didn’t give me any tablet or injection.

Did your friend say or do anything at that time that made it more difficult for you to terminate the pregnancy?

Yes because when we realised that I was pregnant my friend asked me about the plan I had for the pregnancy then I told her, tell you what I have no plan for this pregnancy, I want it terminated but I don’t have the money. Then she asked me; ‘do you want to keep this pregnancy yet you are taking care of these other children single handedly with no support from their father?’

I told her that I wanted to terminate it but I had no money so she responded by saying are you keeping it yet a child with no family is a burden? The child can never understand that you had a problem so you couldn’t know the father, the child will blame you for this. This affected me so much so I told her that I didn’t have any details about this man and honestly I didn’t know because I used to see him at the bar since he was a customer. I didn’t have the chance to know him more and someone who has harmed you in such a way… is this a good person? He is not a good person. Those are the reasons as to why I had the termination. I had a very bad experience but I didn’t regret having terminated it.

You said it took you time to come up with the money, so apart from that what else made it difficult for you to terminate the pregnancy?

Nothing, I was so determined… I wasn’t even bothered by that feeling that I could lose my life, I didn’t mind about the consequences because of the pain I had, I didn’t even think about other people getting to know about it or that I could die, no I thought about all that after the process was done. Before that I didn’t think about anything like other people getting to know and they gossip about me, maybe I would be arrested by the law enforcers, I was not bothered about anything of the sort my only worry that day was the money to pay for the pregnancy termination. I didn’t think about anything else.

So you paid 50,000/=

Yes.

Like you’ve discussed about the whole process, how did you feel about the fact that it is your friend and niece who had to pay for pregnancy termination?

I was affected so much knowing that there are certain people I was burdening, having a challenge and it is these people who have to solve it yet they are not financially well too. Diverting someone from her personal responsibility and this person has to cover my own responsibility, this is not easy given the situation of today’s income levels.

You said that you went to a clinic, so what were the most important qualities in a place and method that you considered when choosing where to go and which method to use? Like you said before that some people use local herbs while others use biomedical so why did you decide to go for biomedical care?

I used the biomedical because it is faster, herbs take so long as you experience a lot of pain. And since I had pain from wounds on the different body parts the three of us had a discussion and decided that I would go to the clinic. They said; ‘’let us work together to save that money so that you receive the service.’’

The health worker told us that even if I don’t take medication provided the fetus comes out then I will have no health problem. That’s how we ended up in a clinic and the gentleman helped us.

How did you get to know about the clinic?

That friend of mine used to work there as a cleaner so she told me that she used to see people being given that service so she told me to go and explain to him. She was once an employee in that clinic.

Before going to that place did you think about the qualities of the place where you were going?

No (laughter), all we wanted was the pregnancy terminated, that is all. You cannot even think about other issues…no.

How about safety?

That I might have challenges? Health worker, you can’t think about that the challenge comes in when the pother process is done for instance when I started bleeding I started thinking; the bleeding is so heavy that I’ve started getting paralyzed. That’s when i was given herbs to help me gain my energy and blood but before that you cannot think about it because of the urgent need at heart.

How do you feel about the way you were treated when you went to have the pregnancy termination procedure?

Health worker, it is a very difficult situation, I had a bad experience while it was being done and when I went back home too so I don’t want to think about it… I had a very bad experience I can’t tell you that the experience was good. I had a bad experience… I had a bad experience to the extent that one time I woke up and I was lying in a pool of blood, the blood was too much… it was too much… I had a very bad experience. ((Tears rolling down her cheeks as she talks))…

Did anyone help you feel comfortable during that time?

My friend supported me because she would come very early in the morning to check on me before going to town for work, she would ask me; how do you feel? But you will be fine. After some days she would say; the bleeding is not as heavy as last week so you will gradually get better, tomorrow I will bring you a certain herb for such an ailment, I will boil it for you to take.

Sometimes she would bring me food in the morning for breakfast on her way to work and she would tell me please eat, don’t wait until you feel hungry to eat, please eat so that you improve. These two people were so supportive, they would come and I talk to them and this encouraged me so much.

How about that day when you went to the clinic for the termination, did your friend support/comfort you in anyway?

Yes she told me that after the pregnancy termination I would be able to resume with work and support myself like I did before but remaining with the pregnancy would affect me. She encouraged me and by the time I went I was so confident with the hope that once this is done my life will be normal again.

1. TRANSCRIPT NAME: *Participant 1_Kampala, age 37*

You have shared with me the process you went through to make the decision to end your pregnancy, what I wanted to inquire, did you know right away, what you wanted to do or did it take some time for you to decide?

When I knew that I was pregnant, immediately my mind started to work, because already I knew that there was nowhere I was taking this pregnancy. It was taking away my peace, so I thought of a lot of things, infact, a lot of things.

If I was to tell you everything! You see that man, I even reached a point where I asked myself whether the pregnancy was really his. Because I’m a SW, you start getting other thoughts and say maybe it wasn’t his, because we stay on these roads and sell ourselves, and condoms bust. So I got many thoughts and said maybe the man knows the pregnancy is not his. I thought of a lot of things; I’m already pregnant, I’ve told him I’m going to remove it, but he replied me that if you want, just remove it. I decided quickly by myself that I shouldn’t take this very far, I will do this and it doesn’t become a problem to me.

So whose final decision was it?

It was mine.

Okay, did you have anyone else to talk to about what you were going through?

That friend of mine whom I have old you about, and she is the one who helped me.

Did anyone else help you make the decision to terminate?

No for sure that was me, I didn’t even need to ask anybody else for their opinion.

You’ve told me that some of the reasons for your pregnancy termination stemmed from the partner you had at the time, so let’s remind ourselves the steps you took to end your pregnancy.

As soon as I got pregnant, I went to the pharmacy. Infact I first bought a pregnancy urine test, and confirmed I was pregnant. I went back and bought the pills, which I used, thinking I had succeeded but unfortunately it was not a complete abortion. Then I shared with that friend of mine because I felt the pain was increasing and I couldn’t even leave the house and so she brought herbal medicine and we cooked it, and after cooking it, I took it.

What is the name of that herbal medicine you took?

We call it ‘luwoko’.

What is it composed of?

It just grows in the bush.

So it is to be cooked?

Some say it kills, but for us we cook it until it’s ready. They say when someone eats it when it is raw, it can kill someone, but for us we cook it, we cook it until it becomes very ready,

‘Luwoko’

Yes, and then we drink it.

How many cups do you drink?

She told me to boil it until only 2 cups of liquid remained, so when 2 cups remained, I drunk them. After taking it, I saw that something had come out, but after it came out, I stayed with a lot of pain, I stayed with a lot of pain.

When you say something came out, how long did that take?

After I had drunk it, some things came out of me, I even pressed myself until I could see that they were getting out, but I stayed with a lot of pain.

So you saw that blood came out?

Yes, clots of blood, so when I saw those big clots, I thought it was successful, but I stayed with a lot of pain. So after some time suffering from pain, I told her although I removed it, I am not well. The stomach pains me a lot, I don’t want to eat, and she gave me wisdom herself, and she told me to go to RHU, and I went to RHU.

What is the timeframe from the time you took the herbal medicine to the time you went to RHU?

Remember I had started in July, but she took me at the end of September, so they first gave me some treatment. Anyway I also got a chance at the time, because at the time when she took me, I found when some whites had come. I think their donors who give them money, and so they found me with my problem and the excessive pain I was in. They talked to me, came to my home, saw my many children, and saw the place I stay in. So I think within them, they got mercy and said that they would treat me. So they paid for me money, took me to the scan, from the scan, they paid for me to be evacuated, and they gave me medicine and I became fine, but even now I still feel the pain inside me.

What were the scan results reading?

In the scan, they told me there were clots of blood, that had stayed inside. So when I brought the results to them, they knew what to do, and they worked on me and I became fine. I wasn’t walking and I started to walk. I wasn’t eating, I started to eat, but I was in a very bad situation.

How did you find out about these places where you went or the methods you used; because you used about 3 methods. You talked of a pharmacy, for example

For the pharmacy, I know about it. Most of us know about the pharmacy, that when I go to the pharmacy, I can get something because already we hear about the pills. A lot of sessions come and the health workers teach us that there are pills.

But they don’t tell you the names of the pills?

They tell us but we don’t take heart to know, and although we don’t know the names, we are told that in the pharmacy they are there, you just know. I also know the names but I’ve forgotten, and I know they are there in the pharmacy. Most times we use them, and sometimes the way they use them is not right and they fail to achieve what they wanted.

Who taught you about the use of these pills?

The health workers teach us even in the past, when you get pregnant and go to the health facility, they tell you how to use them and even give us in the health facility, and we know about them.

I have understood, so for the herbal medicine, it is your friend who told you about that method?

Yes, my friend.

For her where did she learn about it?

She just knows *(laughs)*

Before this, had you known about RHU?

I knew about it, because in this area, I think it is the first organization that we started with and it was also using us as their peers, but just because after some years, the projects end and they let us go.

I still want to go back a little bit to your friend, how did she know the herbs to use?

Let me tell you, most of us know these things.

So when she brought you the herbs

*I knew about them [herbal plants], but I didn’t know where to get them from. I knew there was herbal medicine but the specific type was chosen by her called* ‘luwoko’ *[phytolacca dodecandra] and she brought it for me, but we know these things. There’s another herb called* ‘ennanda’ *[commelina africana] which we insert in our private parts, to terminate a pregnancy.*

Okay, what challenges did you face during the process of getting your pregnancy termination?

So many, first of all I couldn’t make money during that time. The man had left me, I didn’t even have what to feed these children, indeed I was in a very difficult time. Remember these were covid times, and there is no one you could run to, to give you money but you just have to plan. I was treated so badly, and for me if my children have nothing to eat, I don’t have peace.

Remember you are staying in a rented house, you don’t have what to eat, you are not earning. From the 7^th^ month up to the 10^th^ month that’s how I was. To look for money again, I think it was the 11^th^ month at that time. Remember all that time the landlord is demanding and children want to eat, that period was really not good.

And what about your friends, are there things they did that didn’t make you happy during that time?

I really can’t accuse them, because if someone doesn’t stay at my home, they may not know what is happening to me. The only person who knew was my friend, and if she had 5000 shs she would give me.

And what about the health workers?

Only the RHU health workers knew and they knew about my termination after some time really and they really helped me.

Was it a challenge to find a place or choose the right method for you?

No, because Indeed, when I terminated the pregnancy, I didn’t see any challenge because I decided that the pregnancy should be removed and I wanted it removed; I didn’t see any challenge in that.

Did you pay for the pregnancy termination in any way?

No at RHU, I didn’t pay.

How much did the pills you first used cost?

They cost me 3000 shs, they give you 3 pills at 3000 shs, I think each tablet is 1000 shs, maybe also the testing kit. I had bought that at 2500/-.

Did you pay for the medicine you got?

No, I didn’t pay for the medication. For the herbs, my friend just went to the bush, those herbs are available if someone just goes to the bush and searches for them.

And at RHU?

I didn’t pay anything because the whites I found there paid for everything.

It was a chance

Yes, because once they knew what my problem was, I was at home and the whites came, interviewed me on the pain I was in and yet they had also given me the first aid at that time. They had given me some tablets which I was still taking when I saw them bringing me the whites. I had already met them before and they knew the pain I had. They saw me, saw my situation at home, saw the children, the challenges I had, and the next day I was called that the whites had paid money for me. I was told to go to the scan and see what was going on.

For the scan, they put me on a motorcycle and took me to kalwere, and they worked on me and brought me back. The doctor said it was necessary to evacuate me and they did so, and they gave me the medicine.

What are the most important qualities in a place and method that you considered when choosing where to go and which method to use?

When I knew that I was pregnant I’ve told you the situation I was in, I didn’t have money and I said let me go to the clinic so that I can buy those pills.

At the pharmacy

It was a pharmacy.

What qualities did that pharmacy have, why did you choose that place?

Because I know that it sells all the medication one needs, it’s a big pharmacy that has all types of medication.

Was it near your home?

Yes, it is.

Did RHU have the qualities you were looking for?

Yes, they cared for me, and moreover I was broke financially, but they showed me care, indeed they helped me.

How was the privacy at RHU, was it of concern?

Privacy was there. The way they do their things when they are working on you, nobody else can know what is being done. Other people think it is a hospital for other diseases I didn’t worry about that at all.

What about convenience?

They were fast and It was convenient.

How was it at the pharmacy?

I faced no challenge with the pharmacy, they also worked on me quickly. As you know pharmacies, everyone is doing their own thing. One person works on you and another is minding other people or their own business, so they worked on me well.

How do you feel about the way you were treated when you went to have the pregnancy termination procedure or purchase the pregnancy termination medication?

At the pharmacy there was no problem because I went, got what I needed and left.

And your friend who gave you the herbs?

There was no problem with her, infact she knew my challenges and worked towards ensuring that I get better.

And at RHU?

There was no problem there, because they showed me a lot of care, they really cared for me.

Okay did anyone make you feel less comfortable in any of these places?

No.

And how did you feel about the information you received?

At RHU they asked me why I first tried to do it myself, why I didn’t come to them in the beginning, they really advised and counselled me. They told me I wouldn’t have done it that way by myself because I would have died. “Why didn’t you come to the health center and tell us and we help you?” They were not happy that I had used pills and then herbal medicine.

By the way I had not told the health workers at RHU about the herbs I had taken, but when we went to the scan, it showed I had taken them. The person told them that I had tried to terminate and probably used herbs to do so. For me I had just reported that I had swallowed pills, but they later found out about the herbs.

So when you returned to see the health workers after the scan, what happened?

Then I told them the truth, the RHU staff don’t have a scan. So the first time, I went, I just told them I was in pain, I had tried to terminate and they gave me some medication, and as I had told you, I found some whites there. So when they took interest in my case the health workers gave them more information about me, and I went back home. The whites must have pitied me, because the next day the RHU health workers brought them to my home, and they asked me questions and left. The following day the RHU staff called and said the whites were interested in helping me and in paying my bills, but they asked me to come to the health facility, which I did and the rest is history.

1. TRANSCRIPT NAME: *Participant 5_Rakai, age 38*

Now, in our first conversation, you told us that you have ever aborted, which year did you have your last pregnancy termination?

In 2000

When?

In 2020 December.

So how did it start, I would like to know how it was? What did you go through to abort it?

Where I aborted from?

From the time you conceived. Okay how did you know that you were pregnant?

I had a man whom I considered a boyfriend but I was surprised that he had gone, he was from Kenya. I didn’t have his phone contact and address but before he went, he knew that I was pregnant but I came to know about it later that I was pregnant, I was no longer seeing him around, I have this child, I am the father and the mother.

So what I did, *I shared with my friend and she told me about a woman who gives herbs. I took them and by 6pm abdominal pains had started, then after it came out. I saw that something had come out so I quickly to cut it and it fell down then I got up, padded and stayed there for a week. But when I came back, I noticed some things remained inside although I was walking. T*hen I said that let me go to XXXX hospital. *I went to hospital and told them that I had a miscarriage but there are things that remained inside. T*hen they told me that “go for a scan, so I went for the scan and they wrote for me medical forms. You know how hospitals operate, they told me that go there and they clean your womb. When I reached the hospital they placed me on machines and put me on a drip, they wore gloves and pushed their hands inside with a bucket there as they cleaned my womb, then they later injected me and I walked away.

How old was the pregnancy?

It was 4 months old.

Four months? So how did you know that you were pregnant?

Pardon?

What signs were there to show that you are pregnant?

I just realized when I had started vomiting and food started smelling bad, that is how I went to the Musawo who tested me and told me that I am pregnant and it had grown, it was 4 months old.

You hadn’t realized it for the entire 4 months?
 No , I used to move very well but started vomiting when soap started smelling badly, my heart beat had increased and I saw that I didn’t have … then I said that let me go and see a Musawo like I explained to you.

Now, how did you feel when you came to know that you were pregnant?

I lost peace (was uncomfortable) because I have this child who I am taking care of, I am the father and mother, now I have another, will I keep having pregnancies without someone who can stand with me and support me? This means that I will have all pregnancies without any one supporting me? So I lost all my peace, I didn’t have peace.

Isn’t there any one helping with the first child?

There is no one, I am the one taking care of the child , I just saw the father playing cards so I decided to carry away my child and started preparing food in hotels suffering while I was educating my child in baby class to top class. Then the father died and I remained acting as a father and mother and was still, the one who was supporting and educating the child, I got a loan and I ran away from Mbarara and came here.

You ran away from Mbarara because of a loan?

Yes I had 3 loans from Finca, Trust and Brac but when things failed I started working in hotels then officials from Trust Bank came and visited me at the hotel but the owner of the hotel knew my brother so she called him and explained everything about how Trust Bank Officials had come to take me with them. Now because my brother knew the person I was working for, he told them that they give them some 10,000/= but they refused and then they said that they give them more 100,000/= but they refused and wanted to arrest me yet I had paid and was almost completing.

Then the owner of the hotel paid more100,000/= to make 300,000/= so they left me but I still had the FINCA and Brac and that is when I said that let me run away with my child who stopped studying and I came because I was not myself. I managed to survive and then my brother asked me for the other money so I paid him the 300,000/=

The money he had paid for you?

Yes when he helped me, the time Trust bank officials were about to arrest me so I sent it as I continued educating my child until she completed senior six. She completed during the other first lockdown and she started working in Kampala and now she told me that mummy I am going abroad and she went. So I was confused and said “I do not want those things of going abroad there is no peace, then she said that “mummy you would just pray do not say that”. I then said that God you are the one who gave me this child, I cannot keep her so if you have planned to protect her while there then that is okay. So I left her to go, she is now abroad but I educated her out of standing out there in the cold (as a sexual worker)

So she is abroad, in which country?

Saudi Arabia

So the father was not helping during that whole time?

He passed on.

What age was the child when he passed on?

She was in primary two when they called me that bring the child to bury her dad. I even did not want to take her there to burry because of the way I had suffered because of him but then later my heart told me that you take the child because time will come and the child asks me that, “where is my father”? But if you take her there and she sees where the father was put, she will not disturb you in future. In future she may become rich and she feels that she misses her father and she later tells me that “you have been a bad woman why didn’t you show me my dad?” If you take her for the burial she will know that I have no dad .

Okay so you continued suffering with the child?

Yes I suffered with her as I struggled to survive outside there in cold (doing sex wok) and they abuse me that “see that grown up woman, she is old but I would not listen to that because the loan people took everything of mine. When I reached here in Lyantonde, I used to sleep down like … I even do not want to remember that kind of life.

Okay you said that you had a loan in FINCA?

Trust and BRAC.

So how did you leave Mbarara?

It’s my friend who gave me advice after seeing that I was struggling. She told me that” do you know what Grace?” “Go to Lyantonde and stand on the street and do sex work so that you get money and get what to do.” So I also came, became strong and started working up to where I have reached now. That is it.

I heard that those people of BRAC confiscate people’s property.

Yes they give you money in a group of 10. So they took things and shared them.

1. TRANSCRIPT NAME: *Participant 7_Kampala, age 31*

What challenges did you go through during the process of terminating the pregnancy?

Okay, first I felt so bad. I hated myself. I had many thoughts. I wanted to go to bar but I could not. I was not feeling well may be because I was thinking a lot not knowing whether I will be able to terminate the pregnancy. When no one owns up the pregnancy, you end having a lot of thoughts given that you will be responsible for everything.

Is there any other challenge?

No, because after I terminated the pregnancy, I thought it is over.

Is there anything else you used to terminate that pregnancy?

No because the first termination I used machines. However, my friend already knew that women use tablets though she also first used Commelina Africana (a local weed). However, ever since she learnt about tablets, she advised me to use them when I called her. Therefore, I said to myself the tablets are fifteen thousand shillings and we went to buy them. There is no other thing I have used because this one had discovered tablets.

How did you pay that money?

I paid in installments. This is because at that time I was not financially well. However, if you have someone and she knows things about you, you can pay in installments. I had some money but with issues about terminating a pregnancy, I had to ensure that I have money at hand and so I lied that I do not have and she accepted. Therefore, the money I had was to help like buying drinks and eats because after terminating, you need to drink a lot and eating well for you to recover your health quickly. If you terminate without experience, some people may not know what is happening to you. The problem is not having enough drinks because your appearance deteriorates first.

How many have you terminated?

2

Apart from your friend paying the money on your behalf, is there any other way you paid for the tablets you used to terminate the pregnancy?

No

What are the most important qualities that you considered when going to buy the tablets at that place you chose?

I may not know the qualities of the place where we bought the tablets because the only thing, I knew is that the place sells the tablets I wanted yet most other places do not sell them. These tablets are sold secretly because it is against the law.

How about the method you used?

I went for tablets because I never had money and yet it was a quick method and also my friend uses them. When you take at night, the fetus comes out quickly

Did you feel that swallowing the tablets was the safest way?

Yes, because my friend and all the pregnancies she has terminated apart from one she has used the tablets and I learnt that experience from her. She told me never again to get bothered. Even those who ask for one hundred thousand shillings, never again give it to them, seventy thousand shillings never again get bothered. For me I have somewhere I have discovered. Tablets alone are just enough; I can get you those tablets. For me I had not known about those tablets. Therefore, I cannot spend seventy thousand shillings on other methods. Even now if I get pregnant, I just immediately call my friend because at the other place they know her. I cannot just go there to buy because they may not give them to me. May be there is a way she talks to them and they understand such that any person around does not understand what she wants.

You told me that after swallowing the tablets, you experienced some side effects.

*I experienced very bad side effects. If my friend did not come early in the morning, the effects might have worsened…I do not understand these tablets [for medication abortion] because I experienced terrible abdominal pains at night from about 2am, and I thought I was going to die inside the house. But, I had already swallowed them and there was nothing to do. In the morning, I started bleeding very much and I got worried again that I was going to die inside the house, which would be shameful. Then the foetus came out but there was a problem within my uterus, which I did not know about. It was later that I started experiencing more pain. I consulted my friend and she brought ‘Kamunye’* [Hoslundia opposita] *to drink. S*he also advised me to apply pressure on the stomach but she told me that after some time. *However, I realized that tummy was swelling and I told my friend that ‘you know what let me go to hospital because there appears something that might not be right.’ At the hospital, I was told that if the swelling had increased beyond, I would have died. I had clots in various places and so if I had not gone to the hospital, the medical officer told me I would have died because many women have died like that.*

What happened after talking to the medical officer?

Nothing happened after because the officers attended to me and they washed properly inside my uterus, I became fine. They also gave me some tablets to that dries the blood. I also even know these types of tablets. The officers gave them me and directed me what I should do but for me I already knew what I was supposed to do. Because when you terminate, you have to eat well to be able to recover very fast. If you are a worker, you can miss working for two days.

How much did they ask from you at the hospital?

I paid fifty thousand shillings. Even then, I did not have it. However, my friend got it somewhere and paid her in installments.

So, when you got that medicine from that medical officer you got fine.

Yes, I got fine till today.

Now how did you know that the pregnancy termination was complete? You said that it failed the first time, the stomach got swollen and you had to go to the hospital.

The fetus came out, When the fetus comes out because many use it, I have some medicine that we have to swallow and you think that you are fine yet some blood clotted somewhere inside. For me I did realize that there and then. I started noticing my abdomen starting to swell and I was advised to apply pressure on it but there was no change. I had to tell my friend that you know what I have to go the hospital for checkup. What is inside this abdomen, why is it swollen? When I went, they found blood inside and they washed my inside because the uterus had started getting damaged.

Now when you got that last treatment, did you know that everything is out?

I knew that everything is out because I did not experience any other problem until now.

1. TRANSCRIPT NAME: *Participant 17_Kampala, age 24*

Which other person did consult when terminating the pregnancy or which person did you inform when going to terminate?

R: I asked my friend who told me not to terminate.

I: Is there a way it [pregnancy termination] affected your friendship with that friend you asked?

R: Yes, she got upset with me.

I: What of that gentleman who was responsible for that pregnancy, did it affect your friendship with him?

R: We separated

I: Was it because you had terminated his pregnancy?

R: Yes, because I had terminated his pregnancy so we separated there.

I: Apart from him beating you, which other reason caused you to terminate that pregnancy?

R: Sometimes, he would deny the pregnancy saying it was not his, he used to do that. I knew it was his pregnancy.

I: Are there other reasons for terminating pregnancy apart from those two?

R: There is no other reason.

I: How did you discover the place where you bought the tablets?

R: That place… my mother is the one who took me there since she had also terminated a pregnancy from there.

I: Apart from those tablets you used, is there anything else you first used before those tablets?

R: Before we used the tablets… yeah, they were also tablets she gave me; two where put [under the tongue and they melted] and the others were pushed down but termination was unsuccessful the first time.

I: So, you got another place

R: We got another place. That is why the blood which came out had clotted. I think that is what caused the blood to appear like that. The traditional medicine, someone told me [my sister] that I should get omo (detergent), put soda and drink, the pregnancy would get out but I refused. I knew I would die

I: You considered those tablets you swallowed as the right way, since you refused to drink soda and omo knowing it was not good. Where those tablets safe for use?

R: No, *I was afraid of them [tablets] but my money would afford that because the other method was costlier. They were going to suck out the fetus and it cost 100,000/= yet I had only 50,000shs.’*

I: Where did you get the 50,000/= from?

R: My mother gave it to me

I: Did she get it from somewhere else?

R: No, she works

I: Is there any other way you managed to pay the 50,000/=? Did she sell anything in order to get that money?

R: No, she did not sell anything, she has a stall in the market selling tomatoes and onions.

I: Which place was that where you got tablets to terminate the pregnancy and they did not work? Who directed you?

R: My sister first directed me; she was the one I first told when I went home before telling mother. She took me there then we went to the health worker’s home. She has a hospital she works in and it is a government hospital. She gave me those tablets from her home since we were staying near her. The pregnancy refused to get out and she told me not to terminate it saying that it seems at my husband’s side they don’t terminate pregnancies.

I: How do you feel about the way health workers where you went [the first place you went to terminate the pregnancy]? How did you feel about the way the health worker treated you?

R: She was first afraid saying how old is the pregnancy and I answered her. She thought the pregnancy was two months and she said if it makes four months, she does not terminate it. She asked if I was able to insert tablets under then I told her I could not. She said she would put the tablets there and when I go back home, I should take warm water with tea leaves. She talked to me nicely.

I: What about the second place you went to, how did you feel about the way she/he treated you?

R: She did not talk to me badly when we reached her, she asked for the months and a surety. I told her mum is the one standing in for me. She touched my stomach and said it is not yet. She then asked for fifty thousand shillings.

I: At the time of terminating that pregnancy, you were challenged financially, the money was not enough and you tried terminating at first and it failed? Did you get any other challenge?

R: The other challenge I got was being abused. Before I terminated it, looking for money was a problem because it took mum three days to get it. There things I got tired of like the smell of omo and eating offals. Whenever I eat such, I would vomit, my mother would abuse me, plus my young sisters used to say that it is a pitty giving birth from home [okuzalila kulujja]. I had no peace and yet I had run away from the man as a result of beating me. I hated myself which led me to terminating the pregnancy so that I walk away.

I: Does that mean after termination you walked away?

R: Yes, I walked away after, now I live with my friend

I: Which other challenge did you encounter apart from what you have mentioned?

R: The village people talked about me telling me of how I terminated the pregnancy. They said I had terminated ten pregnancies, five pregnancies yet this was the first.

I: How did the village people get to know?

R: The time I fainted people came home due to the placenta retention. I lost too much blood, I fainted and I was taken on a boda boda. That is how they got to know, they came and saw blood in the bathroom. I felt I was ashamed in the village and limited in movement.

I: When you went the second time and they washed your stomach, how did you know it was all done [termination complete]?

R: I asked the health worker if it was done and he said yes. I thanked him then he told me to wait for a period of one year before conceiving. I agreed and he gave me review days. I returned up which she checked and told me all was well. This was done in a government hospital; I did not tell them I had terminated the pregnancy I told them it just got out (it was a miscarriage).

I: If the pregnancy had failed to get out, what might have happened?

R: I would have understood that it has failed, if it happened the second time, I would have believed that what the first health worker told me was the truth. I would have left the pregnancy.

I: Why did the health worker say she works on those with young pregnancies?

R: She said the medicine she has works on those with young pregnancies like one month, two months like that up to two and half. If it is past those months, she does not work on such people.

I: Could that be the cause of your first failed pregnancy termination?

R: Yes, the pregnancy was grown and I did not know. I did not know how to count the months.

1. TRANSCRIPT NAME: *Participant 12_Rakai, age 34*

Which year was that?

Last year (2020). When I came back I told my friend about it so we went to the public hospital and I was given some medicine for these wounds on the arms and on the leg. I didn’t promptly mind about these other aspects but after sometime I realised I wasn’t getting better I had a low appetite. My friend told me but I think you cannot fail to eat because of the wounds, what is it? You have started vomiting too. I told her ‘’I don’t know.’’

This is when she said what if you conceived but I said I don’t know but since am going back to the health facility for wound dressing I will ask them. When I went I had no money so my friend told me that a certain health worker said we can pay 5,000/= for the pregnancy test so my niece gave me the 5,000/= and when the health worker carried out the pregnancy test it was positive.

It was at this point that my friend told me that I have a friend who is a health worker so we can go and explain to him so we went and I explained to him. When I did he asked me what I wanted to do so I told him that; ‘’I need your help to terminate this pregnancy’’. The health worker said ‘’do you have the money?’’ I told him that I didn’t have the money so he said you will have to go and look for 300,000/=, I told him that I didn’t have that money and honestly I didn’t have that money.

What kind of health facility was this health worker working in?

It was a clinic. I told him [health worker] that I didn’t have the money and he asked ‘how much can you afford?’ I told him 30,000/=. He laughed and said ‘I can help you do it at 50,000/= but with no extra medicine given’. So I had to look for the 50,000/= which took time to get because I was not working. It is my friend and my niece who contributed to get the 50,000/=, which we paid to the provider to conduct the abortion, but he didn’t give me any tablet. After the abortion, he said ‘you go and whatever happens do not tell me.’ We went home but I had too much pain the whole night then the fetus came out in the morning, it was about three months old because we took over a month to get the money. The bleeding was too much, I would take boiled herbs, I was so dizzy. My friend would come and buy me millet flour, sometimes cassava flour for porridge, until God helped me to recover.

Does it mean that you didn’t take any tablet?

He didn’t give me any tablet.

How about prescribing for you?

No, he didn’t prescribe any medication for me because he told me, I will just start the process but will not give you any medicine. So I asked him why and he said that I had to pay more 20,000/= for the medicine so I told him I didn’t have that money and when my friend told him that he should give us the prescription he said; ‘’do you want to put me in trouble?’’ So he refused and didn’t make any prescription for us.

That is why I used local herbs only like ekisuula leaves, plus many other herbs, there were some red herbs which are similar to doodo (a type of vegetable) which were boiled for me to drink, I would use some other herbs for bathing. There is another type of herb which helps to reduce the bleeding, it was boiled and I took it too. My friend is older than me so she knew these herbs better than me, she would boil them for me to take and the other types were for bathing. I cured but it took me some time, God had mercy on me so I got better.

How long did it take you to recover completely, no bleeding and you have recovered well?

It took me about three months for the bleeding to stop and feeling better, I wasn’t working, I was limping due to the wounds and this leg was swollen too, I had a sprain, the wounds on the hands too were so painful. Honestly, it was not an easy situation.

How did you resume work?

When I came back the people who used to sell me charcoal before called to inquire why I wasn’t working any more so I told them I owed the land lord some money for rent so they said they would give me the charcoal and I would pay them in installments after selling.

I went to the land lord and explained to her about the accident I had, actually majority know that I had an accident. So I told her that I needed some time to work as I pay for example if I get 20,000/= I pay, I get 40,000/= I can pay her until I settle the bill. That is how I resumed work, I didn’t sell any asset or even get cash from anyone. I would sell one or two sacks of charcoal, I buy food for the children as I save some money.

About getting to know that you are pregnant you said it is your friend who told you that you might have conceived, hadn’t it occurred to you?

It didn’t occur to me because I didn’t know that one could be kidnapped and raped only once and she conceives, I didn’t think about it. Honestly I didn’t think about it I thought it was due to the challenges I had that my health was changing so I told her I think the fever is due to the wounds. I had no appetite, I was vomiting, I had nausea all the time, the time for my periods had passed but I didn’t mind since I usually have a problem of skipping my menstrual periods whenever I have any challenges or stress in life for two to three months.

She asked me if I was having menstrual periods and I told her I wasn’t but it is due to the stress I had caused by the problems I had. I don’t know why but I skip/miss my menstrual periods whenever I have a shock from any challenge, I don’t know why but it happens. So when I told her she said but how about the vomiting? So I said; “no just bring me a herb called kakuba musulo it seems I have fever caused by the wounds”. Then she said; “you might have conceived’’, so when I tested I was pregnant.

How did you feel when you found out that you were pregnant?

Haaa!!! Musawo, it was a difficult moment…it was a difficult moment because I felt so so bad, Musawo I felt so so bad… honestly I cried for two days ((as she cries)) and whenever I think about it I feel so bad, it is a very big burden in my life. I went on overcoming it gradually, my friends too were comforting me… ((Interview paused, respondent in tears))

Okay… am sorry… but good enough it was terminated…let’s focus on the future now you will be fine.

I tried on my own as I thought of how to survive, how to take care of my children.

1. TRANSCRIPT NAME: *Participant 9_Kampala, age 25*

You had hinted on what I am going to ask however, I would like a full description of it. What was the process you went through to make the decision to end that pregnancy?

R: For me when they told me that I was pregnant, the first thing I did not want to be married and I still wanted to study. At that time termination was the solution even though I loved the boy because he lacked the ability to act as the husband and father. If I would marry him, it meant that I had to live with his parents in the village since he did not have a house and financial ability. I would be subjected to that life of digging in the village and not leaving with my boy friend. The second one was I wanted to study and I did not want to get into the childbearing situation when I was still young and I would not have another chance of going to school again because the person who was paying school fees would have abandoned me. Then there was a possibility that after giving birth, the child will be taken away and I will be chased away since that is what happens sometimes. Therefore, the immediate decision that came to me at that time was to terminate the pregnancy which I think is a secret so that I continue staying in school because I could not bear stopping schooling to carry on with it up to birth and then go back to school because there I would no longer such a chance. This is because the person who was paying my tuition would have already abandoned me. I shared that decision with my mother who also agreed to it because she feared to be the talk of members of the community and yet I was her first child from an orphaned family raised by our mother. The talk among community members would be she has raised them in a situation similar to hers and yet most of the children that mother gave birth were girls. Therefore, the solution was to do what we thought was a secret in order for me to continue with schooling. Because when I was expelled from school, I had to seat at home and look for another school.

I: When you decided what did you do?

R: When we made the decision, we looked for money and I went to the hospital to terminate the pregnancy.

I: What happened next? I want you to tell me the whole process.

R: When I arrived at the hospital, we had already made a phone call to a medical officer. Before, it was not in my mind to make a phone call to the medical officer but it was my mother’s idea. May be my mother consulted the man who gave my mother the officer’s phone number. I had to call the medical officer because you cannot just go to the hospital and tell the receptionist that I have come to do this. You do not sit together with other patients and they register you. I came direct when I had already made an appointment with the medical officer who gave us directions through the phone call that we go to the room, which is like this. When I arrived there, the processing involved using machines. It was not that easy because first you become ashamed given that you are like giving birth and yet no child will come out and even those who see you entering that room know it as where pregnancies are terminated. Therefore, if they see you talking to that medical officer, they know that the person has come to terminate the pregnancy basing what they already know what takes place there and your age. When I entered, I went through that processing of inserting those machines in my uterus that made me feel a lot of pain but the officer mocked me that I have to with stand it because it is I who did it and I have to get it over. I went through a lot of pain but I managed to come out and the officer gave me some tablets and I came back home. However, at home, it was too much because at first, the pain was bearable but it increased when it was time for the pieces of what the officer cut inside the uterus to come out. At first, a lot of blood came out and then after blood stopped, these pieces came out one at time until when all of them were out. Every piece that came, *I cried and regretted that I will never do it again. I wished I had given birth. The pain worsened every time I thought about it. During conferences, they advised us not to abort because it is a bad omen and one may never conceive again. Whenever I listened to that, I felt guilty, it would hurt me* so much because I knew whatever they were talking about, I had already done it. *I got scared that I might fail to conceive and I made a promise to only get married after I become pregnant. I wanted to get married after confirming that I can conceive, but I failed my own promise, because I ended up aborting again. Another thing was seeing my breasts sagging while other girls were firm. I felt bad losing my beauty yet I could not tell any of the girls that I aborted.* However, we school girls we tend to have conversions in the dormitories and during those conversations, girls would talk about how they were still virgins and this would hurt me so much to the extent of regretting what I did.

I: Tell me more about cutting the fetus into pieces

R: The medical officer cut it and gave me tabs which he said that will push them out. After 2 days the pieces starting coming but were painful. It was at night when I was in my bed and I realized I was sleeping in something unusual. When I touched it was blood followed by certain things and I would through them in the latrine. That was the method he used not pulling it out at once. And I heard that it is a risky method as some pieces get retained in the body resulting into death. God helped me and all came out

I: Did you go home immediately after the process?

R: Yes

I: The other time we met you said that you slept at someone else’s home and saw the doctor the following morning

R: Eeeh, yeah. We had met him late the first time so he just inserted something in the private parts. He widened my legs and inserted certain things, which I presume was medicine and inserted cotton as well and then told me to go back the following morning

I: you said that when you came from school you thought of terminating whose decision was it?

It was mine and mum bought it because she feared to be talked about. I was the first born

I: Tell me more about the boy’s reaction after informing him that you were pregnant

R: first he forced me to have it(sex) I had told him that for me I would only do it after getting married but he lured me into having it. As s.4s are leaving school they love those things of last touch and that was the root cause. I had refused because I had never done it. He kept saying that I can do something to you that is regrettable, truly he did it. He would call all the time to inquire if I am ok and I think he was already aware of what he had done so when I told him what we had confirmed his response was very bad and I felt so bad since it is not what I expected. He said that do you think it is I going to take care of you. When I got a chance of terminating, I hated him so much, I destroyed everything he had given me. I hated men with all my heart but after one year I regained my feelings for him because I truly loved him. We revived our love and ended up in marriage

1. TRANSCRIPT NAME: *Participant 15_Rakai, age 18*

In this community, which term do you use to mean a pregnancy termination?

We say that we are going to terminate a pregnancy/abort. Others say we have gone to remove it *((okuwokonkolamu)),* actually they are many terms.

Which terms?

Usually we commonly say I have gone to abort. And if you are going at least you inform one person you trust. For instance, your parent, a friend whom you trust or your sisters but you cannot tell someone you don’t trust.

What term should we use which is not offending to you?

To terminate a pregnancy.

That is what we should use?

Yes

Do you have any other terms you use?

No

Would you say that pregnancy termination is very common among your friends or your relatives?

I do not know many people who had a pregnancy termination. I don’t know anyone who had a pregnancy termination.

How about among your family members, would you say that it is very common or not so common?

It is not so common.

Not common among friends or family members?

It is not very common among relatives and friends.

How does a person find out that there was a pregnancy termination among the friends or family members?

When I have not disclosed to them? They can notice that you have lost weight in case you didn’t have good care. They can notice that this one had a pregnancy termination if they had seen you pregnant before so they can know that you had a pregnancy termination, because a woman who has terminated a pregnancy can be noticed.

How is she noticed?

You can notice her Musawo, depending on her usual behavior, the adults can easily notice our condition, that so and so is pregnant. Then when she finds that you have changed your behavior then she can know that you terminated it.

And then she gets to know?

Yes, she will know that you terminated the pregnancy even if you don’t tell her and keep it a secret, adults can easily know about it.

Is there any other way they can find out?

There is no other way they can find out when they have not noticed you unless the health worker who attended to you has disclosed it. Apart from that, they cannot know unless your parent informs them and actually a parent cannot call for something bad to her children.

You said that unless someone tells you or when they have seen your appearance

If at all she has noticed before that you are pregnant and then she then your behavior changes from what you had before, then she can know that this one had a pregnancy termination.

Has anyone ever told you about it that ‘you aborted’?

Yes, a certain gentleman in our village told me, he told me that, “you are deceiving you are not sick, you are not suffering from fever, you say the truth that you had a pregnancy termination, someone can tell, you are not like that’ usually”. They told me that I had a pregnancy termination.

Are there women who came and told you so?

Not as such

I was asking about the women individually talking to you, that for me I had a pregnancy termination?

No

Why do you think women can decide sometimes to disclose to you that “I had a pregnancy termination?”

In case I trust you then I can tell you but you cannot disclose to a person you don’t trust. People are rumor mongers, one can know and then tell the other, then also that one will tell another person who will also know and in the end you have lost your dignity. Even the man who would have married you gets to hear about that, even if he is to marry you...

Does she inform you simply because she knows you also had a pregnancy termination or?

Unless you are friends, and she told you when she had a pregnancy termination, then in that case I can also tell you. If you are friends then each one of you has got another person’s secret. If we happen to quarrel and you spill out my secrets, I do the same too.

Is there any other reason why one can say let me inform so and so? Do they have other reasons why they inform another person?

She may not have trust in the people she lives with, then I can maybe talk to my elder sister, in case I don’t trust my mother I can talk to a neighbor and I don’t tell my mother. Well, it can happen.

Okay, it comes from trust. I am very much interested in what women in your area where you live talk to each other about. What do they talk about as women?

In the first place us women are rumor mongers, wherever you find women they are usually backbiting others. The moment she understands that you had a pregnancy termination you become a topic for discussion. Inside our gate, there are women only and all of them are married but you become a topic of discussion the moment they know that you had a pregnancy termination. The fact is that they know I had a pregnancy termination and I also know that they had a pregnancy termination there is no one who can talk about one another.

Okay, in such a situation.

Yes.

Okay, which other information do you share among your friends while seated together as women, it could be your family members? What information do you usually talk about or share?

You cannot fail to get them.

Right, but what is that information, you could be in the salon? What do women commonly talk about?

We usually converse that so and so did this, the other girl down there failed to get married, things like that. For us women we are good at rumor mongering we can never talk about developmental issues, and it is horrible if a woman is talking about fellow women, we like rumors.

How about issues concerning friends in their homes, jobs, education, don’t they talk about such issues?

They don’t usually talk about it, I usually don’t talk to them, you know my husband refused me from associating with them, he refused, and that is why he doesn’t like me to move around.

Apart from rumors and discussing about those who have failed to get marriage, what else do you talk about?

Again, they talk about their husbands, the man did this and that, like that.

1. TRANSCRIPT NAME: *Participant 18_Rakai, age 33*

I would like you to tell me, what those issues are, you point out that such and such issues I don’t share them.

The health worker I talked to yesterday, asked me whether I was currently living with a husband or partner. I answered no, then she asked me why? I told her that the time I got pregnant and later aborted, the man who was responsible for the pregnancy was a foreigner, the man was Kenyan in Eldoret. When I left after conceiving that pregnancy which I was not ready for, I returned and immediately aborted since it was the reason why I returned. I had gone for work so when I learnt that I had conceived I returned.

When I knew that I have aborted and it is now over, I thought that let me plan and go to Saudi Arabia for work, so as a prerequisite for those supposed to go there, you shouldn’t have HIV, one should not be HIV Positive. I think the person who impregnated me is the same person who infected me with the disease because before I left I was working with Dreams Project and Rakai Project and they used to test us frequently, they were not working with infected persons but when I returned I was pregnant at the same time I went on to test when I am HIV positive.

So I cannot share such conversations with my friends, I keep strong and endure the pain heart and feel hurt inside me, and the health worker advised me I should share it with any health worker.

Whom can you disclose such information to?

I have to talk to the health worker or counselor where I get my drugs from at Uganda Cares, I talk to them because they are counselors. I have not yet got a partner because I am not sure whether he is infected or not, I wouldn’t like getting involved with someone, according to the advice the health worker I talked to yesterday gave me that he will be invited we have a discussion he is tested so that we get to know the truth about his status. This is because I may fear him yet sometimes he could be infected too, those are the issues I don’t discuss with people.

Do you have any other issues you don’t share?

Other issues I don’t share are after aborting that pregnancy, you know I used traditional herbs I eventually got a very foul smell from my private parts and it took some time, as if that wasn’t enough much as the abortion was complete I got a discharge, it doesn’t smell nor itch but I have it and I can’t disclose such an issue to people, I keep quiet and suffer in silence.

Do you still have it?

Yes, I still have that discharge but it started from the time when I had an abortion, after the abortion I bled heavily, then afterwards I got too much yellowish discharge flowing, so that thing continued up to now, actually for me I cannot use a white panty, it doesn’t smell or itch but it remained flowing and I cannot tell people about that, I keep quiet and keep suffering maybe I can disclose to people like you who came to me.

If a woman aborts who should know about it?

A person to know about it should be your best friend, a friend indeed.

If she aborts what kind of people should she tell?

A person I should tell, I didn’t tell anyone apart from one person, if you happen to talk about that incidence it might spread yet it’s not good, I have to inform only one person and also keep it a secret. With carrying out an abortion you expect to die and in case you don’t die …, for me I trusted only that one person I told. Well with me after I had overcome it, “I am not dead” although I still have this problem I called my sister and told her that the condition was going through was a result of my carrying out an abortion because I knew my sister cannot betray me and spread the information to the people around. In most cases it is not good for that information to be spread around, and someone says; you see that one she aborted.

I would like you to share with me your experience from the time you learnt that you conceived to the time when you had the termination.

I got pregnant in November 2018 and when I learnt that I was pregnant I started planning how to end the pregnancy, I tried to go to the health facility but they were using Swahili, there was no way I could explain it to them. From that time I started devising means of how I could return home. I had gone to work in someone’s home and it was in that home where I found that man who impregnated me, then I returned here in December.

I left as if I had come for a Christmas holiday but my mission was that I was not going to return to that place, I didn’t come with anything apart from the money I came with in December. I came and decided to hide for some time at my friend’s house until the end of December, I conceived in November, moved on to January and terminated it in March 2019.

Did you abort from your friend’s home where you stayed?

No, I carried out the abortion from our village deep, deep in the village at a certain woman’s place in our home where I was born. I used to walk in the night because I had not even told them that I had left the other place, I came back and hid myself at my friend’s place in Kakoma then I began making calls in the village seeking information to know the time when she would attend to me because I didn’t want to stay there for long. This is because some other people who know me could come to do the same thing and find me there, so I used to hide, I could travel in the night to that place called Gayaza.

Were you using motorcycles?

From where I had hidden to Kyotera I could use a taxi, then from there I could use a motorcycle but this motorcycle I could use was not supposed to be owned by people from my home village, I tried so much to get a motorcycle rider from Kyotera. I didn’t go there once, I went there the first time to inquire then she asked me about the pregnancy duration and I told her that it is coming to four months. She examined me and said it is true it is soon making four months.

She said, “I charge depending on the period of the pregnancy, I don’t charge the same amount for a one month pregnancy like a two months’ pregnancy”. The amount of money she wanted me to pay at that time was too much, it was 100,000/= yet I had come all the way from Eldoret so I spent too much on transport. I told her I would return in two months’ time so I came back this side.

Later on I decided to go back with 70,000/= I begged her, “please help me I wouldn’t like it to go beyond this period” and she accepted. She told me, “drink this, sit on this one, you should squat so that it can enter inside you. Then she told me to return to this place because the next stage…, she asked me, “do you live with anyone? I told her yes I do, what is the relationship with that person, is it a friend? I said yes, it is my friend. She said okay, this and that will happen. She then gave me cotton, she always has it that it’s included in the charges. She told me you will feel that thing coming out, if it comes out you can go to the toilet, you can use a bucket when it comes out. But I really felt pain!! I even regretted why I had taken the herbs, my dear “nalajana” *((literally meaning that she cried out in pain)),* I had great pain, “nalajana”. Suddenly I felt as if I wanted to pass out urine, I felt something so I squatted to urinate then it came out.

But still, after the fetus coming out I continued having problems, blood didn’t stop flowing, I continued bleeding, I lost weight, I lost weight, it continued flowing and later I had a bloody discharge, I could not move to any place due to the bad smell, I could smell it too, I lived with it.

After taking the herbs, how many days did this great pain last before the fetus came out?

Hope I have not forgotten, she gave me the medicine on Wednesday, no, no, it was a Thursday because she said she does work on a Wednesday, it’s a day for what…, the traditional healers know how to explain what it is. She gave it to me on Thursday and I took it, then she gave me herbs for bathing plus other pounded herbs which she told to me to bring and put them in a basin or polythene bag and sit in it.

With water?

Yes, mix it with water in a basin and sit in it. So I took it on Thursday, then on Friday at night, I first took from her place and also sat in it, she again gave what I should sit in when I reach home, but she never gave me more for drinking, so I brought it and sat in it from this side. Then on Friday at night, I felt pain up to the extent of tying up a cloth around the waist but I think it was towards morning, it’s when I got that great pain, big clots of blood came out, clots of blood came out, clots of blood. Some were black so I thought could be it is because I drunk those herbs, could it be the foetus which was developing, that is how it was.

But the pain took almost two weeks, I was in pain at the same time bleeding, I was feeling pain at the same time bleeding but I had nothing to do only that I was sure I have aborted it because ever since I conceived I had not seen blood again.

Didn’t you go back so that she could stop that pain?

No, after that time I was using my own knowledge, you know I had given birth to a child in the past, so I could go to the bush and my friend could get me herbs, it is called “Ekisuula” it cleanses in the stomach/uterus ((*kwoza mu lubuto)),* I used to take that Kisuula, I continued taking it.

Did you boil it?

Yes I used to boil and take, I never went back to her because what I wanted was to abort, I aborted and didn’t die, then I said let me just use this to cleanse inside, like a woman who gave birth normally, so I continued doing that.

However, similar pain to that of a woman who has given birth went on for almost two weeks, even within those two weeks blood kept on coming out but it was not as much as that one which came in the beginning. The discharge kept on running and I had to keep on padding myself, I could feel myself smelling badly, later this foul smell stopped and I remained with the discharge running up to now.

1. TRANSCRIPT NAME: *Participant 6_ Kampala, age 25*

Apart from her saying you will not deliver and whose money are you wasting, what else could have forced her to tell you to terminate the pregnancy?

She knew I was to further my studies and that is what I thought too. We reached a time when the examination results were out to go to university yet the place she was hoping to get money was no more. It was literally impossible to get money

Did she tell you where she got the money to buy the tablets she gave you?

She used to work

What was her work?

She used to have a stall in the market where she was working.

According to the way mum gave you local herbs to terminate the pregnancy, did you feel that was the right way of terminating? How did you feel inwardly when they brough the herbs?

According to me “musawo” as I am telling you the truth, I would not want someone to terminate a pregnancy in whichever way unless if they say that the pregnancy will cost her life. Like some people are told they have a medical condition but if it is not a medical condition, I would not like it because at the end of the day, that child can grow and benefit the world or you benefit as a parent. This is regardless of the age at which you have delivered that child because I know God makes no error. God can give you that pregnancy or he can stop that pregnancy. God never gives you a child in error; I do not encourage anyone to terminate a pregnancy personally. At this point even when I get pregnant for another man who is not the one with me, I deliver that child reason being that is a child who is blameless.

Now, taking you back to the other question. How did you feel about the method of termination that mum used to terminate the pregnancy?

It is not good at all; reason being you can die based on the way I was feeling for the first three days, truthfully it is not good.

Apart from the way, you felt weak and failing to get out of the house, what exactly did you feel during that time?

The stomach pained me too much as you hear it actually affected me because after senior six my aunt got me a man in 2018. I was with that man but I could not conceive until 2019 when I conceived. I think it brought complications because after that at the end of 2018, the man did not know though he gave me money to go to hospital and they had to first clean my stomach with those machines. They had to clean my stomach since I would get complications, as I would bleed smelly blood even after the termination. My mother allowed me to get married saying that the man would help me. My husband did not know and all he was aware of was I bleed smelly blood and he gave me money to go to hospital. I kept on tell him that my stomach had to be cleased and still he was not aware.

Why did aunt bring you a man?

That is how our aunt behaves; you know things in the village. When she gets a man from Kampala, she tells him of her daughters in the village. She then comes to pick you up whether you have appreciated or not you just go.

Did you appreciate the man?

Ok… musawo he is not too old and he is around. It is not that I appreciated but just that I was tired of being home. The truth is that I was tired of being home.

What made you get tired of being home?

There are so many things; mummy would abuse you all the time in line with the mistake I made. She abused me the whole of senior six even when I came back home for third term. She was saying that I think you have brought another pregnancy, but I think we shall have to deliver that one but I would keep quiet saying no word so I got tired of home. When aunt brought in that suggestion, *I said am off the rope* let me go regardless of what I will find there whether it will be good or bad I will run away. I said good enough I have entered Kampala I found shelter there.

Is that the same man who fathers the twins?

Yes, he is the one

What if that pregnancy did come out successfully, what could have happened?

Schooling would have stopped at that and I think I would not have sat for senior six. I would have ended there but the child would be alive. I had plans to work because I had my sister who was in Kampala and would tell me that any time after school I would come. I think that would not have happened; I would first raise my child that is what I know.

Do you have anything else to say concerning that pregnancy, anything we could have forgotten?

The owner of the pregnancy run away when he was told that I was pregnant. He run way from the village and I have never heard from him.

Were you from the same village?

Yes, we were

How old was he?

He was also schooling

Up to now, you have never heard from him?

I do not want to hear from him.

1. TRANSCRIPT NAME: *Participant 3, Kampala_age 28*

How have your pregnancy terminations affected your relationships with friends, family or people you know? Which effects have these abortions had on your family? Is there any way it has brought any changes or any effects to your relationship with your friends or your family?

No! There is none. It has not had any effect.

Is there no effect? That I was hated or that I was chased from family?

No! They never chased me! No! There are no effects. There is nothing.

Who supported your decision to have a pregnancy termination?

Which one? The last one?

The first and the last one!

For the first one, the one who supported me was my boyfriend. I was in support of it as well. For this last one, it was my friend who supported me.

Who opposed it?

No one, because for the one I told, she first discouraged me and later she told me that “my friend, if you will hustle, you keep it”. That is how it was.

Now talking about the issue of stigma, can you describe any stigma surrounding pregnancy termination?

In which way?

The way they are treated. The women; can you tell me the bad ways that the women who decide to abort are treated. What we would refer to as stigma! A woman has decided to abort and then people begin treating her badly when they realize that she aborted. It can be people in the society or from her family. Have you heard of such stories?

People…

[interjects] and they begin treating you badly because you aborted! Like we talk about stigma with AIDs. When people know that you have AIDs, they begin segregating you. Now when we come to abortion, does it also come with stigma?

It’s there. It’s so much because when one knows that you aborted, they begin showing dislike pointing at you saying that “that one aborted”. And also, there are those people who abort like the sex workers down there in ‘Kimombasa’. They even abort mature pregnancies or they can produce the baby and then they suffocate it and then they dump it in the trench.

So, when they get to know about you, you would have brought a very big stigma in the area only that some women hide because after doing it they first disappear. Even recently, there is someone we did not know who gave birth to a baby and she suffocated it. I think she tried to abort and the baby came out.

Like how old was the pregnancy?

It was like 7 months old. I even have a photo with me here. I don’t know whether I can show it to you. Is it not necessary? It was about 7 months and she dropped it in the trench. And if they realize that it’s you, who did it, like you know stigma issues, they will point you out. They can even beat you up.

Now this issue of stigma like you have explained it to me, how did you go through it? Did you ever get stigma? Did you get people who discriminated you or who abused you after knowing that you had aborted?

Like I told you that the person who came to know about it was one! I never talked a lot and I never got big stigma. But I myself put stigma on me. I was not myself among the people, I wasn’t myself where I was sitting thinking that maybe they will get to know that I aborted. I stigmatized myself. I think that is how it was.

Did you have to hide your pregnancy termination from anybody?

I had to hide my dear!

Who did you hide from?

I hid from some of my friends and also my neighbors. I hid from them apart from the one I told you about whom I shared with.

Why did you hide yourself from them?

Now for all of them to know that I aborted?

Umm!

Yes! I feared them to circulate that information around and to stigmatize me.

Now for those people you told; that friend of yours you shared with; did you feel that you were helped? Were there good results after telling them? In other words, did you feel relieved?

I got much relief because at first, she did not support me. But afterwards she called me and said that “do you know that you have aborted 2 but I have aborted 4?” So, she gave me courage and I was like I aborted the first one and I recovered and my friend has also given me courage. I got relieved and I became strong.

If you were to abort another time, would you have shared with the same people? Would you tell that friend of yours?

No! I cannot tell her again. I would tell another person like my sibling.

Why?

She may say that “will you be aborting every time?” Ok, it depends because then she was close to me and now, she is a bit far. But if am to abort again, I tell my sibling or any other person, like only one!

Now my last question!

Yes!

What would have happened if you were unable to have a pregnancy termination?

*Ha. I don’t know what would have happened because I was finished. I was going to hustle with that pregnancy yet I had no help. I already have two other children here and they are still young and yet I am the father and I am the mother at the same time. I would be pregnant without any help. Let me not lie to you, it would have been so bad*

Ok!

Yes, I would not manage!

If you were not able to abort this pregnancy, what else would you have done?

If I was not able to abort! Of course, the other thing I would do was to give birth but doing so through suffering and troubles since I would be carrying a baby I never desired and not ready to have. Right now, I am suffering with these other children and I again bare another one even with out a person taking care of it.

So, if you were not able to abort, how would life have changed or how would it have been affected?

I would be suffering, let me tell you! I would be suffering with it. I would have to look for some thing to eat yet I am pregnant and again have the other children. No! It would have been bad. W*hen I aborted and returned in my normal state, I began working for these other [three] children, because I am the mother and the father*.*.*

How many children do you have?

Three!

Do you live with their father?

No, my dear! I live with my children!

As we are winding up, are there some other things related to this conversation that we have not talked about and you would like to talk about them? About the abortion conversation! Is there any other thing that you wanted to share with me or talk about?

What I would like to talk about; ok like us, we need to go ahead and sensitize women on how to abort so that they don’t abort using; thinking that they will drink ‘*ennanda’* or thinking that they have the knowledge that; let me drink ‘*ennanda’*. We have to teach them…

[interjects] who gives them that advise?

They get advice with in themselves. We would have taught them so that they know about safe abortion or teaching them about family planning most especially the young women because they are not confident. You will teach her and tell her that go for family planning and she will lose confidence of going there because she will meet boys there. You may think that this is a young girl yet she has more than one man she is sleeping with.

Ok!

Yes! So, for me all I say is persistent sensitization of women both old and the young ones who fear. Because, I even have a girl who recently; the girl is my neighbor and I have been seeing her. She is like 17 years but she is ever in so much company of boys and roaming around.

So, I sat her down and I counseled her that “you would have done like this and that” and that “you use family planning”. So, she showed me that she has no boy she is having sex with. Right now, she is pregnant. But I tried to sensitize her and she showed me that she has no boy she is sleeping around with but right now, she is pregnant and even her parents came to know about it. That’s it. Maybe when you have another different thing you are asking me about.

Unless you feel that in your story, there is something you have not told me; even about terminating a pregnancy.

Even about terminating pregnancies! I don’t have much I have not told you. May be this method of using tablets, and also the method of internal cleansing or MVA,

Yes!

The difference there is that MVA; because I have also ever used it, is a bit more expensive than the one for tablets.

How much does it cost?

It depends where you have gone. It’s more expensive! Also, another difference with it, they wash you and you go away when it has been removed, when they have completely washed it out better than the tablets where you swallow and you go and it aborts from home. For it, they wash you internally and you go away when it has been removed. But it is also painful, it’s really much more painful than the one for tablets.

1. TRANSCRIPT NAME: *Participant 21, Rakai_age 38*

We are now almost coming to an end and you told me about this but let me understand it better, for the last pregnancy that you aborted, did you only tell one friend about it?

Yes that one I told because you cannot talk much and people know that you aborted and they imprison you. So for such a thing you only have to tell one person about it. The one you know and trust.

But you told her after the first thing had come out?

Yes I told her after that “I didn’t see the after birth”. Then she told me that go to hospital those things can kill you.

Is that the very woman who directed you to the herbal lady?

Yes.

So when it came out and some remained in, you again told her?

Yes. Then she told me that go to hospital.

So do you feel that sharing with this friend helped you?

It really helped me.

How?

It helped me because she told me to go to hospital so I had to listen to what she told me to do because we started this thing the 2 of us.

Why did you particular talk to that friend of yours?

It’s because she is my friend so I had to tell her from the beginning and in the end I also told her because she was my friend. I could not tell anyone else so it’s her that I had to tell.

Why couldn’t you tell others?

Are they your friends to tell them that? You tell your friend whom you know that they cannot tell anyone else.

Now had did sharing with this friend of yours about the abortion affect you? Okay how did you feel after sharing with her?

You feel good because when something is on your heart and you share it, your heart feels support and you relaxed. You can tell her that I feel that this has hurt me and she tells you that do like this and you also remain thinking about what you had shared with her when you are a bit relaxed. It’s better than sitting alone in a room, quiet, you have things you are thinking about but if you have someone to share with and you say that let me call this friend of mine and I share with them. So after you tell her, you share ideas and when she goes, you now start having another plan.

Okay but you feel telling your friend helped you.

It helped me a lot.

Is there anything negative that came out of it or that did not make you happy?

There was nothing.

So if you to abort again would you share it with her?

She is the one I tell.

Why?

It’s because when the other one happened it ended there it was spread .

It didn’t spread?

No

Okay that’s good. Now what do you think would have happened if you had failed to abort?

And remained with it?

Yes

Of course I would be suffering, I would be like a mad person because you do not have the Childs father and even the first child is in the same situation and so it means that even this one is in the same situation. You will find that in the world, you are just like …you won’t be able to buy a mattress, you won’t buy smearing oil, you won’t buy clothe because all the children are your responsibility. How do you think you would feel? Won’t you look like a mad person? Haven’t you seen people who have clothes, has no soap, no because of the bad situation because all the money will have gotten finished on those children but if you have one and you are taking care of him, you will take care of them slowly by slowly, if he says he doesn’t have something like food, you can but it slowly by slowly because of your earning because there is no one in the background helping you.

So tell me if they are 3 or 4 how will you feel? Won’t I be like those on family planning…has failed, it’s not easy Musawo it’s not easy. Children are good but they are good when you are 2 people, you keep contributing, this one brings posho and you both struggle to get school fees, if this one pays you can also buy a book. The children need when people are two but when you are alone, you will break a lot if they are many. If I had not aborted the other one would not have studied.

You struggled, you struggled …

I struggled and even the girls said that “eeeh Grace you struggled. “everyone says that, “Grace did it, Grace you did it because I used to sleep on the floor because the things were taken so when I came I was down there , so the girls said that “ Eh this fat lady sleeps on the floor” they used to talk ,talk but they didn’t know that I was paying school fees for my child.

So when the situation started getting better and I bought saucepans and other things, I picked the child from my brothers place and brought the child because he wanted me to cook for him food, people saw a child coming back from school dressed in uniform then they said Grace is not easy, she … yet she educates her child and ever since then people started loving me because they saw a child going to school, they said, “This woman suffers because of her child “ever since then people started liking me because of educating the child but before that they were wondering where I put the money that I get and every one could speak what they want but for me I used to save it because I had to take the child back to school every term.
