# Supplementary material for: Lived experiences and drivers of induced abortion among women in central Uganda
Source: PLOS Glob Public Health. 2023 Dec 6;3(12):e0002236. doi: 10.1371/journal.pgph.0002236 (PMC10699625; doi:10.1371/journal.pgph.0002236)
Supplement: S1 Table — (DOCX) [file pgph.0002236.s003.docx]

**S1 Table: Checklist for Consolidated criteria for reporting qualitative studies (COREQ)**

| **Item Guide questions/description** |  | \| **Location in manuscript.** \| \| --- \| |
| --- | --- | --- | --- |
| **Domain 1: Research team and reflexivity** |  |  |
| ***Personal Characteristics*** |  |  |
| 1. Interviewer/facilitator: Which author/s conducted the interview or focus group? | SPSK, SN, GKN supervised data collection | Data collection, page 6 |
| 2. Credentials: What were the researcher’s credentials? E.g. PhD, MD | PhD, MPH, BA. | Author details and data collection, page 6 |
| 3. Occupation: What was their occupation at the time of the study? | University Senior lecturer, Professor, Project Social scientist, Research Scientists, Research Associate |  |
| 4. Gender: Was the researcher male or female? | Data collectors were female, supervised by female and male | Data collection, Page 6 |
| 5. Experience and training: What experience or training did the researcher have? | Minimum of 5 previous data collections for the research assistants. | Data collection, Page 6 |
| **Relationship with participants** |  |  |
| 6. Relationship established: Was a relationship established prior to study commencement? | Yes. Through phone calls at screening for recruitment and informed consent form |  |
| 7. Participant knowledge of the interviewer.  What did the participants know about the researcher? e.g. personal goals, reasons for doing the research | Participants had interacted with research assistants during the survey, before agreeing to the In-depth interviews on another day. Informed consent form and the recruitment script included the purpose of the study. | Design and sampling, Page 5 to 6.  Ethics, page 5. |
| 8. Interviewer characteristics: What characteristics were reported about the interviewer/facilitator? e.g. Bias, assumptions, reasons and interests in the research topic | No interviewer related bias existed. A values clarification training was conducted. | Data collection, page 6 |
| **Domain 2: study design** |  |  |
| ***Theoretical framework*** |  |  |
| 9. Methodological orientation and Theory: What methodological orientation was stated to underpin the study? e.g. grounded theory, discourse analysis, ethnography, phenomenology, content analysis | A qualitative description, with phenomenology leanings.  Deductive and inductive approaches were used to code the data | Design and sampling, Page 5.  Data management, page 7 |
| ***Participant selection*** |  |  |
| 10. Sampling: How were participants selected? e.g. purposive, convenience, consecutive, snowball | IDI participants were purposely selected from a survey sample. | Design and sampling, Page 5 |
| 11. Method of approach: How were participants approached? e.g. face-to-face, telephone, mail, email | Recruited through telephone, and then interviewed face to face. | Design and sampling, Page 5 |
| 12. Sample size: How many participants were in the study? | 40 participants | Design and sampling, Page 5 |
| 13. Non-participation: How many people refused to participate or dropped out? Reasons?  Setting | None |  |
| 14. Setting of data collection: Where was the data collected? e.g. home, clinic, workplace | Preferred place by participant; at home, and private space away from parent community | Ethics statement, page 4 |
| 15. Presence of non-participants: Was anyone else present besides the participants and researchers? | None | Ethics statement, page 4 |
| 16. Description of sample: What are the important characteristics of the sample? e.g. demographic data, date | Average age of 28 years, majority in union, had a child before the most recent abortion. Majority had attained secondary+ education | Table 1, page 7 to 8. |
| ***Data collection*** |  |  |
| 17. Interview guide: Were questions, prompts, guides provided by the authors? Was it pilot tested? | An IDI guide with topics including how information regarding abortion is spread within social networks, decision to abort, and experiences with the most recent abortions throughout the process.  The first interview for each interviewer reviewed to make improvements to the interview tools, especially to the probing questions. | Data collection, page 6 |
| 18. Repeat interviews: Were repeat interviews carried out? If yes, how many? | No |  |
| 19. Audio/visual recording: Did the research use audio or visual recording to collect the data? | Only audio recordings | Data collection, page 6, and  Data management, page 7 |
| 20. Field notes: Were field notes made during and/or after the interview or focus group? | Yes. And these were used in the debriefing meetings |  |
| 21. Duration: What was the duration of the interviews or focus group? | IDIs lasted 1 hour and 12 mins. | Data collection, page 6. |
| 22. Data saturation: Was data saturation discussed? | No. |  |
| 23. Transcripts returned: Were transcripts returned to participants for comment and/or correction? | No |  |
| **Domain 3: analysis and findings** |  |  |
| ***Data analysis*** |  |  |
| 24. Number of data coders: How many data coders coded the data? | Two people |  |
| 25. Description of the coding tree: Did authors provide a description of the coding tree? | Yes, Coding process provided under data management.  And the resultant thematic areas in the conceptual Framework. | Data management, page 7.  And Figure 1, page 9. |
| 26. Derivation of themes: Were themes identified in advance or derived from the data? | Both deductively and inductively derived. | Data management, page 7. |
| 27. Software: What software, if applicable, was used to manage the data? | Atlas.ti 9 | Data management, page 7. |
| 28. Participant checking: Did participants provide feedback on the findings? | No |  |
| ***Reporting*** |  |  |
| 29. Quotations presented: Were participant quotations presented to illustrate the themes / findings? Was each quotation identified? e.g. participant number | Yes | Results Chapter. From page 7 |
| 30. Data and findings consistent: Was there consistency between the data presented and the findings? | Yes |  |
| 31. Clarity of major themes: Were major themes clearly presented in the findings? | Yes, major themes provided in the results and discussed. | Results Chapter. From page 7 |
| 32. Clarity of minor themes: Is there a description of diverse cases or discussion of minor themes? | Yes. Minor themes provided in the results and discussed. | Results Chapter. From page 7 |
