# Supplementary material for: Lived experiences and drivers of induced abortion among women in central Uganda
Source: PLOS Glob Public Health. 2023 Dec 6;3(12):e0002236. doi: 10.1371/journal.pgph.0002236 (PMC10699625; doi:10.1371/journal.pgph.0002236)
Supplement: Attachment — Submitted filename: Response to reviewers.docx [file pgph.0002236.s004.docx]

13^th^ July 2023

Sreeparna Chattopadhyay, Phd
PLOS Global Public Health

**RESPONSE TO REVIEWERS PGPH-D-23-00643**

Dear Dr. Sreeparna.

Thank you for your positive review of our manuscript titled “Lived experiences and drivers of induced abortion among women in central Uganda.”

We have addressed both the journal and reviewers’ comments and are pleased to re-submit the revision. Please see specific points below. Thank you for your consideration.

**Journal requirements:**

The completed PLOS’ questionnaire on inclusivity in global research is attached. The list of legends for the supporting information files have been included.

**Reviewer #1:**

This is a very pertinent study areas and a cause for avoidable morbidity and mortality in low-and middle-income countries. The study has unearthed various drivers for induced abortions, and provide practical policy direction to address this global public health issue. However, in line 281, the spelling for "could" should be corrected. In addition, the authors should include possible areas for future studies.

***Response:*** Thank you for this encouraging comment about the importance of the study. The correction has been done. We provided implications of the findings from line 724.

**Reviewer #2:**

1) "...the authors insist on the restricted legal environment for abortions in Uganda, without mentioning the state of application of the Maputo Protocol, even though Uganda has been the 28th signatory of this protocol since 2010, which is dedicated to the promotion of maternal reproductive health, in particular liberalization and access to safe abortion. It is, therefore, necessary to succinctly demonstrate the existing gap and the barriers to the complete application of this protocol according to the country's context."

***Response:*** Thank you for this important comment. Indeed article XIV, part 2c of the Maputo protocol positions that “States Parties shall take all appropriate measures to: protect the reproductive rights of women by authorising medical abortion in cases of sexual assault, rape, incest, and where the continued pregnancy endangers the mental and physical health of the mother or the life of the mother or the foetus.” The guidelines in Uganda as indicated in this paper do recognise the legality of abortion the same circumstances as provided for by the Maputo protocol. We have added this to the manuscript from lines 69 to 74 to re-affirm that Uganda follows the protocol. However, the lines that follow indicate the potential barriers that may hamper access to safe abortion care.

We have also added this to the discussion (see line 700 and the following) where a clear information gap exists, contrary to what is provided for under the Maputo protocol.

2) "....should provide the necessary explanations in the technique of sampling to justify such a repartition between the greater Rakai (21 respondents) and the city of Kampala (19 respondents) when Kampala is far more populated than Rakai".

***Response:*** Thank you for this. We have added the clarification in the methods section. From line 128. We aimed to balance the two strata (study sites). However, following daily research team debriefings, we noted thematic saturation by the 18^th^ respondent in Kampala, hence the slight imbalance between the two strata.

3) "...data need to be presented better. The percentage gives a rough estimate of the coverage of the variable in the total sample. The author should therefore add a column for percentage in Table 1".

***Response:*** We have added the column for percent in table 1 and highlighted some of the percentages in the text, which we have also modified to suit the updates.

4) "The partner's participation, perception, and opinion are critical in explaining and interpreting abortion drivers. It is desirable to know to what extent the respondents expressed themselves on the support or not of the partner in relation not only to the announcement of the pregnancy but especially to the decision to abort in the participants' general characteristics "

***Response:*** Thank you for this comment. Indeed, it adds further clarity to how big this challenge is. We have added the magnitude of this in the results, under the drivers of abortion (from line 217), where 13 women (32%) reported partner denied responsibility or were not interested (7%). We believe that his is a finding that belongs here, other than the participant characteristics.

5) "The extract from the interview chosen by the author on financial drivers does not correspond to the theme but rather shows rejection on the partner's part after the pregnancy announcement, without emphasizing the financial problem as a motivation or consequence of the abortion. The same applies to the extract chosen for the personal drivers, where the participant, although still at school, seems to have been somewhat influenced by her partner, the teacher".

***Response:*** Thank you for the suggestions. In the first case, we have replaced this with a quote that touches both cases better. From line 270 onwards: “*The truth is that I did not have enough money to care for it, so I decided to abort. But, before then, my heart would say ‘please leave it’, yet it continued to grow and the man’s manners continued to worsen. Actually, he eventually switched off his phone.”*

The second quotation has been replaced with a more direct one: from line 280. *“Sincerely I did not want the pregnancy right away, because I did not wish to get pregnant that time. I think I would have strangled the baby myself after birth”*

6) "The author failed to effectively demonstrate how the community drivers, particularly religion, culture, and tradition, influence the issue of abortion in the result. These factors play a significant role in shaping attitudes toward the topic. Clarification is needed."

***Response:*** Thank you for the very important comment that adds value to the discussion. We note that the issue of religion did not feature in the women’s explanation of the drivers. However, culture and traditions can be interpreted from the results. We have added demonstrated these in the discussion, because the results are reporting women’s accounts and analyzed manifestly. Below are the areas we have enhanced:

The patriarchal system that emphasises the sense of belonging in the community tagged to the paternal side could have driven abortion where the men denied responsibility. See line 616 and the following.

The traditions around the high expectations placed on children has also been added to the discussion, as one of those that may drive abortion. See line 636 and the following

We have also added the culture of protecting the culprits of defilement especially when it’s a family member, as a potential driver but also may minimise the support received post abortion. See line 642 and the following.

We also do point the expectations from the religious perspective to carry the pregnancy even in cases of rape, incest or defilement as impacting on the decisions to abort but also complicating the post abortion experiences. See line 649 and the following.

Thank You,

**Simon Peter Sebina Kibira**

Corresponding Author
