# Supplementary material for: Lived experiences and drivers of induced abortion among women in central Uganda
Source: PLOS Glob Public Health. 2023 Dec 6;3(12):e0002236. doi: 10.1371/journal.pgph.0002236 (PMC10699625; doi:10.1371/journal.pgph.0002236)
Supplement: Attachment — Submitted filename: Response to reviewers.docx [file pgph.0002236.s005.docx]

10^th^ August 2023

Sreeparna Chattopadhyay, Phd
PLOS Global Public Health

**RESPONSE TO THE EDITOR’S COMMENTS FOR PGPH-D-23-00643R1**

Dear Dr. Sreeparna,

Thank you again for providing us with another opportunity to perfect our manuscript. We have addressed the comments you raised and are pleased to submit the 2^nd^ revision. Please see responses to specific points below. The tracked changes version of the manuscript details the edits. Thank you for your consideration.

**Editor’s comments:**

1) The qualitative description [25] on 108 which the paper is *bases* had phenomenological [26] overtones, and included 40 women (19 109 in Kampala city and 21 in greater Rakai areas [based not bases]. Also, the term phenomenological overtones need clarification. Phenomenology is a methodology. What do you mean here?
***Response:*** Thank you for this correction. The statement now reads as follows: “The qualitative study on which the current paper is based used a phenomenological design

to study lived abortion experiences among 40 women (19 in Kampala city and 21 in greater Rakai areas).”

2) You have mentioned a team of 4 RAs collected all the data - are they included as authors? Without their labour, this work would have been impossible. I would urge you to expand the author list to include them in the manuscript.
***Response:*** Thank you for this concern. We respectfully note that the RAs do not meet the ICMJE authorship guidelines. Their role was only to collect the data. We have updated the acknowledgement section, and added the names of the four RAs who participated in data collection. It now reads; “We also thank the research assistants who collected the data; Resty Nakayima, Prossie Aliwebwa, Sheila Kisakye, and Margaret Nansubuga.”

3) There are extensive copyediting and grammatical issues with this manuscript. Please make sure it is grammatically correct before submitting the final version. I have given some examples below but it is not my role to be a copyeditor. I am an editor.

***Response:*** We do apologize for the typos and appreciate your comment to improve the manuscript. We have carefully reviewed the manuscript to correct grammatical errors, and repetitions.

4) "Most women reported that their male partner was vividly not caring or supportive when informed" Please rephrase, clunky sentence.

***Response:*** Thank you. This is corrected. It now reads: “Most women reported that the male partner was not supportive, when informed about the pregnancy.”

5) " One woman was still in a grieving period, just days after losing their partner, and got pregnant with a brother-in-law who offered ‘support’ during this time." Why their since it refers to a woman?

***Response:*** This has been edited from ‘their’ to ‘a’. The sentence now reads as follows: “One woman was still in a grieving period; just days after losing a partner, and got pregnant with a brother-in-law who offered ‘support’ during this time.”

6) "Other reasons included the fear of disappointing parents among young people still living with their caregivers’, a stressful work environment that was not ideal for pregnancy and fear of losing their job as a source of income, among those engaged in work." Why caregivers? And why an apostrophe after caregivers? Shouldn't it be parents or families? Caregivers is used as a term for people who are sick or with disabilities and the reference doesn't seem to suggest that is the case.

***Response:*** Thank you for this correction. It now reads: “Other reasons included the fear of disappointing parents or guardians among young people still living with their guardians”

7) "Although the availability of equipment was not always known, they used indicators like the size of the facility to denote assume they had equipment. "Denote, assume? which one?

***Response:*** Thank you for this. It was an oversight. We meant to replace denote with assume, but didn’t remove the former. It now reads. “Although the availability of equipment was not always known, women used indicators like the size of the facility to assume they had equipment.”

Thank you.

**Simon Peter Sebina Kibira**

Corresponding Author
