# Supplementary material for: Lived experiences and drivers of induced abortion among women in central Uganda
Source: PLOS Glob Public Health. 2023 Dec 6;3(12):e0002236. doi: 10.1371/journal.pgph.0002236 (PMC10699625; doi:10.1371/journal.pgph.0002236)
Supplement: Attachment — Submitted filename: Response to reviewers.docx [file pgph.0002236.s006.docx]

1^st^ September 2023

Sreeparna Chattopadhyay, PhD
PLOS Global Public Health

**RESPONSE TO THE EDITOR’S COMMENTS FOR PGPH-D-23-00643R2**

Dear Dr. Sreeparna,

Thank you again for providing us with yet another opportunity to perfect our manuscript. We have addressed the comments you raised and are pleased to submit the 3^rd^ revision. Please see responses to specific points below. The tracked changes version of the manuscript details the edits. Thank you for your consideration again.

**Comments:**

1. You have mentioned that the study uses phenomenological design. The characteristics of phenomenological research includes a deeper engagement with perceptions, memories, affect and imagination and a sample size of 10-15. Of these your study only uses perceptions and experiences. In-depth interviews of the format conducted here doesn't automatically qualify it for a phenomenological approach. The way in which this data is presented is a straightforward qualitative approach that uses thematic analysis. You need to alter the language and the terms to reflect that this is the case in this study.

***Response:*** Thank you so much for your detailed education on this. We do appreciate you. We are sorry that this line remained in the paper in the previous version. We have made an edit to limit this to a straight forward qualitative approach. It now reads; “The current paper is based on a qualitative approach to study lived abortion experiences among 40 women.”

2. Also the quotes should be followed by a description or interpretation. This interpretive approach is completely skipped in some parts and included in other. Please rectify this.

***Response:*** Thank you for this comment. We respectfully note that the findings are not intended to be interpreted in the results chapter, but to provide a description of the accounts of women. We do provide in the discussion section some interpretation/understanding in relation to other studies as well. We respectfully request to keep the results section as is to avoid addition of subjective statements.

3. Challenges expressed during and after abortion: While the rest of the themes have sub-themes nested within it, this is one long section without themes. Please break this up into sub-themes that come up in the analysis.

***Response:*** Thank you for this comment that helps us to provide more clarity to the findings. We have provided the sub themes in the section as follows: Physical challenges, Financial challenges, Psychological and emotional challenges.

4. The discussion section is well-written.

***Response:*** We appreciate this comment, and are glad to know.

Thank you again for the opportunity.

**Simon Peter Sebina Kibira**

Corresponding Author
